# *fourSig*: a method for determining chromosomal interactions in 4C-Seq data

**DOI:** 10.1093/nar/gku156

**Published:** 2014-02-20

**Authors:** Rex L. Williams, Joshua Starmer, Joshua W. Mugford, J. Mauro Calabrese, Piotr Mieczkowski, Della Yee, Terry Magnuson

**Affiliations:** ^1^Department of Genetics, The University of North Carolina at Chapel Hill, Chapel Hill, NC 27599, USA, ^2^Carolina Center for Genome Sciences, The University of North Carolina at Chapel Hill, Chapel Hill, NC 27599, USA and ^3^Lineberger Comprehensive Cancer Center, The University of North Carolina at Chapel Hill, Chapel Hill, NC 27599, USA

## Abstract

The ability to correlate chromosome conformation and gene expression gives a great deal of information regarding the strategies used by a cell to properly regulate gene activity. 4C-Seq is a relatively new and increasingly popular technology where the set of genomic interactions generated by a single point in the genome can be determined. 4C-Seq experiments generate large, complicated data sets and it is imperative that signal is properly distinguished from noise. Currently, there are a limited number of methods for analyzing 4C-Seq data. Here, we present a new method, *fourSig*, which in addition to being precise and simple to use also includes a new feature that prioritizes detected interactions. Our results demonstrate the efficacy of *fourSig* with previously published and novel 4C-Seq data sets and show that our significance prioritization correlates with the ability to reproducibly detect interactions among replicates.

## INTRODUCTION

The organization of chromosomes in 3D space has a significant impact on mammalian gene expression (1). Variations of the chromosome conformation capture (3C) technique have demonstrated that, while regulatory elements and their target gene promoters can be separated by large linear distances (measured in base pairs) (2), chromatin looping can modify the physical proximity of regulatory elements and promoters to alter gene expression (3–5). The interaction between promoters and regulatory elements can occur within a few kilobases (kb) (6) to several megabases (Mb) (7) on the same chromosome, or even on other chromosomes (8). In addition to bridging genomic loci, chromosome folding is thought to be vital in concentrating RNA polymerases and transcriptional regulators (9). Known to occur for all three RNA polymerases, these ‘transcription factories’ contain multiple active loci and may be a strategy for efficient transcriptional regulation of coregulated genes (10,11). Similarly, correlation of genome-wide conformation maps with gene expression and chromatin modification data demonstrates that transcriptionally inactive loci also tend to preferentially associate with each other, perhaps preventing undesired gene expression (12). Taken together, these data provide support for the hypothesis that 3D chromatin organization, among other functions, influences gene expression by altering the spatial proximity of distal regulatory elements relative to the genes they regulate.

Circular chromosome conformation capture with sequencing (4C-Seq) is a variant of 3C technology that identifies the range of genomic interactions formed by a specific locus (e.g. a gene promoter) (2,13,14). Currently, only a few algorithms exist to analyze 4C-Seq data (13,15,16). To avoid effects due to technical error, such as amplification bias, some methods use data reduction techniques that result in a decrease in resolution, while other methods determine interactions without using such strategies. When capturing genomic interactions, 3C-based techniques do not discriminate between functional contacts and contacts occurring by random chance at the time of fixation. Therefore, during sequence analysis, it is crucial that true signal is efficiently distinguished from background noise.

The currently available 4C-Seq analysis methods vary in the treatment of raw data and strategy for determining signal from noise. Further, none provide a methodology for predicting interactions that are likely to be reproduced among experimental replicates. To this end, we have developed a suite of software called *fourSig*, a conceptually simple yet powerful statistical method for analyzing 4C-Seq data. *fourSig* can analyze data from a wide variety of 4C-Seq protocols, uses the full range of quantitative information captured by 4C-Seq, provides variable parameters to adjust for desired resolution and offers a method of peak prioritization to categorize statistically significant genomic interactions.

Using previously generated 4C-Seq data sets, we demonstrate that *fourSig* is as accurate and precise as the most sophisticated existing methods, but also prioritizes the statistically significant interactions. To demonstrate further the high resolution and effectiveness of *fourSig*’s prioritization feature, we generated and analyzed a novel allele-specific 4C-Seq data set. We hypothesized that if long-range interactions are a general property of transcriptional regulation, 4C-Seq performed at the transcription start site (TSS) of a gene that demonstrates an allelic expression bias may reveal allele-specific interaction profiles. Such an analysis would shed further light on the relationship between genomic interactions and gene expression that is independent of sequence variation.

*Ibtk* encodes a tyrosine kinase inhibitor that displays strong allelic expression bias in F1 hybrid trophoblast stem (TS) cells. After using *fourSig* to analyze allele-specific 4C-Seq data from the *Ibtk* locus, we validated our method by performing fluorescent *in situ* hybridization (FISH). While both *Ibtk* alleles have similar interaction profiles, we identified several interactions unique to each allele using the high-resolution analysis offered by *fourSig*. Interestingly, one allele-specific interaction formed predominately by the active *Ibtk* allele overlaps with a putative enhancer element. Taken together, our data demonstrate the utility, accuracy and precision of *fourSi*g.

## MATERIALS AND METHODS

### Determination of significant enrichment and peak prioritization

*fourSig* is a software suite, written in Perl and *R*, used to identify statistically significant interactions from 4C-Seq data. 4C-Seq reads are initially mapped to the genome. Mapped data are then transformed into a database of genomic fragments using samToRetab.pl or bowtieToRetab.pl (Supplementary Figure S1). Each database is specific to the design of the 4C experiment. This database of genomic fragments corresponds to the restriction site locations of the enzyme used to generate the 3C library (the template for the 4C library). The mappability for any given 3C fragment is then determined based on several factors: the location of 4C restriction sites (both 4C and linearization), the minimum and maximum size selected for the sequencing library, the length of potentially captured fragments, the length of the primer used for amplification and the length of the sequencing reads. After the template database is produced, the mapped reads from the 4C-Seq data are assigned to generate a file that serves as the input file for the *fourSig.R* program. By default, *fourSig.R* removes fragments marked as unmappable within the input database files. Any type of 4C-Seq data can be transformed into *fourSig* format, and a detailed description of the data structure may be found in the Supplementary Methods.

*fourSig.R* allows the user to define portions of the *cis*-chromosome to be masked out before determining significance thresholds. The region close to the viewpoint will contain a high concentration of reads per fragment and will typically require a higher significance threshold than more distal regions. Therefore, this feature may be used to focus the significance testing to viewpoint-proximal regions. Alternatively, regions located near the viewpoint can be masked out to allow for a lower threshold for significance for regions further away. Additionally, a combination of masked analyses could be used to approximate varying thresholds in *cis*.

**Figure 1.**
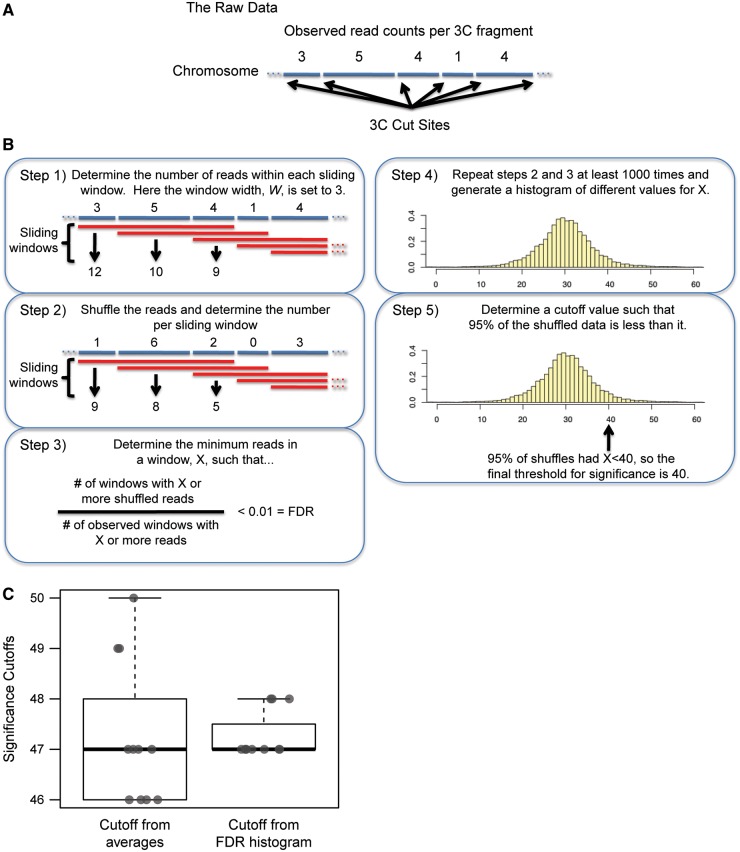
A method for determining significant interactions. (**A**) The sequence of captured fragments is mapped to the genome, and the number of reads mapping to each 3C fragment is used as the observed distribution. (**B**) (Step 1) Sliding windows of a desired number of 3C fragments, *W*, are demarcated, and the total reads in each window are determined for the observed data. (Step 2) The reads on the chromosome are distributed randomly among 3C fragments and a new reads per window is calculated. (Step 3) A cutoff, *X*, is calculated for a desired FDR using the shuffled reads per window data. (Step 4) *X* is calculated from at least 1000 random shuffles to generate a histogram of possible cutoffs for the desired FDR. (Step 5) The final threshold for calling a significantly enriched window in the observed data set is set at the 95th percentile for calculated *X*s. In the depicted example, a desired FDR of < 0.01 leads to a final threshold of 40 reads per window for calling significant interactions in the 4C data. (**C**) Ten significance thresholds were calculated independently using an FDR cutoff based on average randomly permutated distributions (left) and a cutoff determined from confidence bounds applied to a distribution of FDR calculations (right). The y-axis represents significance thresholds in reads per window. Both calculations represent an FDR of <0.01.

The significance testing itself is described in Figure 1 and in the text. In short, a threshold for significance is calculated for each individual chromosome using several user-defined input parameters. The reads on each chromosome are randomly associated with a specified window size of 3C fragments from that chromosome. *fourSig.R* then calculates the significance threshold, the minimum number of reads within a window required to achieve a false discovery rate (FDR) that is defined by the user. The shuffling and threshold determination are repeated as many times as desired; however, we recommend at least 1000 permutations. Finally, the user can specify the percentile from which the smallest calculated threshold that occurred (we recommend the top fifth percentile) is used as the final cutoff for significance for that chromosome. Contiguous windows that are significantly enriched with reads are then merged into a single interaction, and 3C fragments on the 5′ and 3′ ends of each domain that do not contain reads are removed. The analyses described here calculated a significance threshold from the top fifth percentile of 1000 calculations of FDR < 0.01. Parameters for background masking and window sizes varied depending on the analysis and are described in the text below.

**Figure 2.**
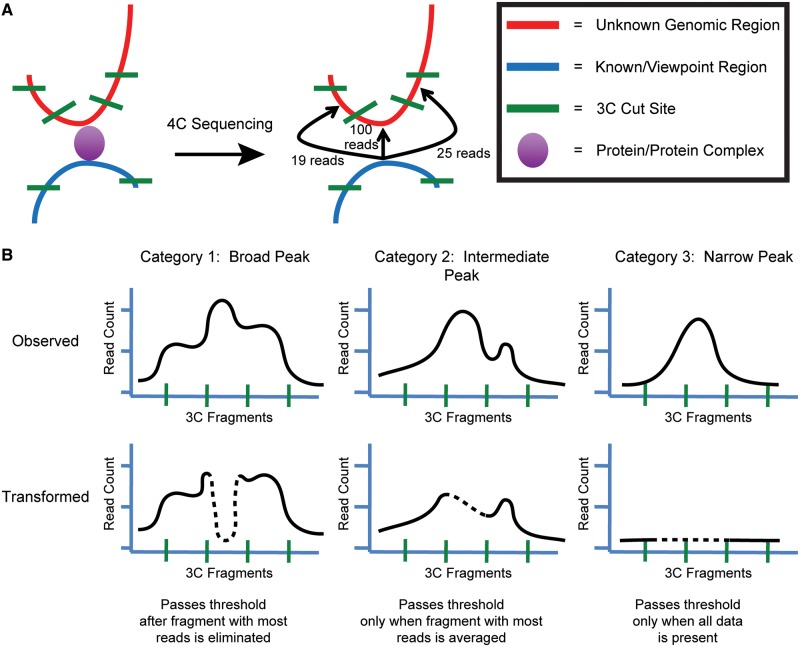
Prioritization of interactions by distribution shape. (**A**) A model of a conformation capture event is shown (left) with a symbol legend (right). A frequently occurring interaction is expected to yield a distribution of reads mapping to locally available fragments and centering on the point of highest probability of contact (center). (**B**) Peaks are assigned a priority level corresponding to expected distributions by reassessing the capacity of a window to exceed the significance threshold on transformation of the read counts for the most abundant fragment (dashed line). Windows are categorized as Broad (Category 1) if the window is still significant when discounting the number of reads in the most abundant fragment, Intermediate (Category 2) if the threshold can be exceeded when the most abundant fragment is replaced with the average number of reads for the adjacent fragments, and Narrow (Category 3) if the significance threshold can only be exceeded when all data are included.

Additionally, we used *fourSig* to prioritize interactions based on the likelihood that they are reproducible. This method is applied to the windows that comprise an interaction, and the entire interaction is categorized based on the broadest assigned designation. As explained in Figure 2, we use an algorithm to manipulate the read count from the fragment in a significant window containing the most reads and reassess the capacity of the read counts from the remaining fragments to exceed the threshold. This approximates the expected distribution of Broad and Narrow interaction categories and allows increased confidence in reproducibility when focusing primarily on interactions designated as Broad.

### Tissue culture

TS cells were cultured as previously described (17,18). TS cells were removed from feeder cells and grown independently for two passages on gelatin-coated plates before sample collection.

### Allele-specific quantitative reverse transcriptase polymerase chain reaction

RNA was harvested from TS cells using the TRIzol reagent and the manufacturer’s procedures (Ambion), treated with DNase I and purified using the protocols described for the RNeasy Mini Kit (Qiagen). RNA preps were quantitated using a Nanodrop 8000 (ThermoFisher), and cDNA samples were prepared from 250 ng RNA using SuperScript III Reverse Transcriptase (Invitrogen). Quantitative polymerase chain reaction (qPCR) reactions were performed on a Bio-Rad CFX96 Real-Time PCR Detection System (Bio-Rad). Details of the *Ibtk* verification may be found in the Supplementary Methods.

### Generation of 4C libraries

Batches of 10-cm^2^ plates of TS cells grown independent of feeder cells for preparation of 4C libraries were treated with 0.25% trypsin to form single-cell suspensions. Plates were pooled for fixation with 1% formaldehyde, counted by hemocytometer and stored at −80°C in aliquots of 3 × 10^7^ cells.

The protocol for generating initial 3C libraries and subsequent 4C libraries was adapted from previously reported studies and was modified for optimal use in TS cells (2,12). 3C libraries were generated by initial lysis, quenching of sodium dodecyl sulfate by TritonX-100, restriction digestion with HindIII (NEB) and proximity ligation with T4 DNA Ligase (Invitrogen). Additionally, a portion of digested preps were withheld from ligation to serve as no-ligase controls. Re-ligated libraries and controls were purified by reversal of cross-links and proteinase K digestion, followed by several rounds of phenol–chloroform extraction and ethanol precipitation. Resulting samples were quantitated using the Qubit Assay Kit for dsDNA (Invitrogen). Effective re-ligation in the 3C libraries was verified by comparing electrophoretic migration in 0.8% agarose with no-ligase controls, as well as by amplification of a ligation product of two adjacent fragments in the *Gapdh* locus (Supplementary Table S1, data not shown). Test amplification of 3C libraries, no-ligase controls and bacterial artificial chromosome (BAC) ligation controls was performed using the SsoFast EvaGreen Supermix (Bio-Rad), and digestion of amplified product by HindIII.

4C library templates for *Ibtk* were produced by digesting purified 3C products with NlaIII (NEB), performing a second proximity ligation, linearizing by digestion with NsiI (NEB). Similar to the 3C libraries, efficient digestion was tested by electrophoretic migration in 0.8% agarose. The *Ibtk* 4C libraries were amplified with Pfx polymerase (Invitrogen) using a double-amplification strategy with primers designed to capture an informative single-nucleotide polymorphism (SNP) on the NlaIII side of this digestion scheme. The primers for initial amplification are homologous to both CAST and B6 sequence and contain a portion of the sequencing adapters (Nextera V1 Universal for NlaIII side and Illumina TruSeq Indexed for HindIII side) with four random bases separating the adapter from the genomic sequence (Supplementary Table S1). Absence of amplification was observed in source 3C libraries and the 4C no-ligase controls to ensure faithful amplification of the intended target. All amplifications were performed within a linear range, as determined by an initial test with qPCR.

After the initial amplification, *Ibtk* 4C intermediates were purified and size-selected (200–650 bp) by gel extraction. Size-selected intermediates were then amplified with Phusion polymerase (NEB) using primers containing sequence homologous to the portion of the sequencing adapter used in the first round of amplification and the remaining sequence needed to complete the sequencing adapters (Supplementary Table S1). Final products were purified using Ampure XP beads (Beckman Coulter) and quantitated using the Qubit Assay Kit for dsDNA before submission for sequencing. 4C libraries were submitted to the UNC High-Throughput Sequencing Facility for paired end sequencing using 2 × 150 bp on the Illumina MiSeq platform (Rep 1) and 2 × 100 bp on the Illumina HiSeq2000 platform (Reps 2 and 3).

A stepwise detail of our 4C protocols can be made available on request.

### Sequence alignment

The C57BL/6 (B6) genome sequence was taken from the mm9 genome assembly for *Mus musculus*, and a genome sequence for CAST/Eij (CAST) was generated by editing appropriate nucleotides with SNPs for *Mus musculus castaneus* (19). Custom Perl scripts were used to handle raw sequence for identifying allelism of fragments and aligning captured sequence to B6 and CAST genomes using Bowtie v0.12.7 (20). Further explanation of the alignment and allelic assessment may be found in the Supplementary Methods.

### Allelic interactions and set analysis of *Ibtk* 4C

After significant interactions are assigned, allelic calls are made based on whether the number of SNPs in SNP-containing reads detected in the interaction is significantly higher than what would be expected by random chance. Chromosome 9 interactions lacking sufficient SNP coverage to make an allelic call were assumed to occur in *cis*. The proportion of Chromosome 9 interactions identified as occurring in *trans* (opposing allele) was used to determine that the likelihood that this assumption leads to erroneous results is negligible (see Results).

The positions of detected interactions were cross-referenced with fragment read data to transform the interaction data into congruent coordinates between replicate libraries for set analysis. Library intersections were determined using the VennDiagram package for *R* (21) to match fragment coordinates. When generating intersection lists of interacting fragments, the broadest interaction classification (Broad > Intermediate > Narrow) was retained as the interaction’s assignment.

### FISH validation

Probes were fluorescently labeled using the BioPrime DNA Labeling System (Invitrogen). *Ibtk* transcript was detected using a cDNA probe covering ∼1 kb of sequence that was generated from a mass-weighted pool of intron-skipping RT-PCR products. Fosmids (BACPAC Resource Center) were used to probe DNA at the sites of predicted interactions and the region immediately upstream of the *Ibtk* gene (Supplementary Table S2). Typically, the *Ibtk* cDNA probe was labeled with Cy3-dCTP (GE Healthcare), the fosmid for the *Ibtk* TSS probe was labeled with Alexa Fluor 488-dUTP (Molecular Probes) and fosmids targeting predicted interactions were labeled with Cy5-dCTP (GE Healthcare).

Details of the FISH procedures may be found in the Supplementary Methods. Z-stack images were taken in grayscale at 63× using a Zeiss AxioImager M2 equipped with an AxioCam MRm camera (Carl Zeiss). Merged Z-stacks were deconvolved using the iterative algorithm in the AxioVision software package (Carl Zeiss). Distances were measured in three dimensions from the approximate centers of DNA-FISH signals using the ZEN 2011 black edition software package (Carl Zeiss). Recorded measurements were used to produce cumulative distance plots with *R*. To produce representative images, z-projections for each color channel were produced and exported with AxioVision, then merged and pseudo-colored with Adobe Photoshop CS5.1 (Adobe Systems).

## RESULTS

### Method for statistical analysis of 4C interactions

#### Determination of significant interactions

4C libraries are generated from downstream processing of 3C templates and are used to identify genome-wide interactions with a fixed locus, referred to as the viewpoint (Supplementary Figure S2). The end product from a 3C preparation is trimmed by restriction digestion, self-ligated and linearized. Although these processing steps generate many molecules, a certain proportion will consist of viewpoint sequence flanking an unknown sequence that was ‘captured’ due to a genomic interaction at the time of fixation.

The creation of 4C libraries inevitably generates certain technical limitations that must be considered when analyzing 4C-Seq data. First, the absolute resolution of the assay will be limited by the frequency of the restriction site for the enzyme used to create the initial 3C library. Second, the choice of enzymes used can result in fragments that are too large to sequence or too short to map to the genome or will be cut in such a way that prevents PCR amplification. Third, because chromatin is essentially a polymer in confined space, the probability of contact will generally be higher for loci that have a shorter linear distance (base pairs, bp) separating them (22,23). This has been observed consistently in chromosome conformation experiments and has been substantiated as a general property exhibited in the identification of topologically associated domains and the fractal configuration of chromosomes in the nucleus (12,24,25). It follows from these properties that in a 4C experiment, one will detect a higher level of background surrounding the viewpoint than at other regions of the chromosome. As such, it would be useful to adjust background thresholds across the lengths of chromosomes. Furthermore, it is expected that if two loci are interacting in a functional manner, then their neighboring sequences will also have a higher chance of interacting. Importantly, these expectations would not be found at interactions occurring by random chance. Taking these issues into consideration, we designed a novel analysis algorithm for 4C-Seq data, *fourSig*, to determine significant contacts with the viewpoint and to emphasize interactions that are more likely to be consistent among successive replicates.

Before statistical analysis, *fourSig* uses a variety of information, including the size of the 4C primers; locations of the 3C, 4C and linearization enzymes; and the size range of sequenced molecules, to determine which 3C fragments can be identified by 4C. Fragments that are too large for the sequencer, too small to be aligned or would not have been amplified by PCR are flagged such that they can be eliminated from the analysis (see Supplementary Methods). This allows *fourSig* to model accurately the random distributions used for threshold calculation (described below).

The schematic in Figure 1 describes our method for determining whether a signal is significant relative to background. Reads are first mapped to the appropriate 3C fragments (Figure 1A). To determine the significance of a contact, we use a sliding window analysis to avoid introducing arbitrary window boundaries. The size of the windows, *W*, can be altered depending on the desired resolution of the contact map. Smaller window sizes provide higher resolution results, which are useful for investigating the details of specific interactions, while larger window sizes are better for showing general trends and identifying highly reproducible interactions (described later).

For each chromosome, the total number of reads in each window is tallied to generate an observed distribution (Figure 1B, Step 1). We then generate a randomized distribution by shuffling the observed reads among mappable 3C fragments. Because a higher background is expected around the viewpoint, *fourSig* allows for the regions surrounding the viewpoint to be treated separately (see below). The total number of shuffled reads per window is then tallied (Figure 1B, Step 2). The randomized data are used to calculate *X*, the minimum number of reads required for a window to be significant by satisfying a desired FDR (Figure 1B, Step 3). The final cutoff value for significance of any given chromosome is calculated from a minimum of 1000 permutations, resulting in a distribution of *Xs* that can be used to define a minimum read count to qualify a window as significant (Figure 1B, Steps 4 and 5). The permutations empirically derive a distribution for *X*, which is used to choose a threshold in which we have high confidence (see below). Using the example demonstrated in Figure 1, 99% of the *X*s from shuffled reads were >10, so it is likely that a cutoff as low as 10 reads in a window would be an underestimate of the true threshold for FDR < 0.01. Similarly, 50% of the shuffles-generated *X*s were >30, so it is likely that 30 would also be an underestimate. However, only 5% of the shuffles generated cutoffs >40, so we can be fairly confident that this threshold does not underestimate an FDR < 0.01.

#### Comparison of methods for calculating FDR thresholds

The *fourSig* method was heavily influenced by a previously described 4C-Seq analysis method (13). However, on extensive trial use of this program, we identified several opportunities for improvement that served as chief motivating factors for the development of *fourSig*. Among these was a considerable amount of variation in the calculation of FDR and subsequent thresholds for *trans*-interactions in repeat analysis of the same data. Using a publicly available 4C-Seq data set (26) and the program described by Splinter *et al.* (13), we performed 10 separate analyses for data on Chromosome 5, each with 1000 permutations, resulting in four different significance thresholds ranging from 46 to 50 reads per window (Figure 1C). The average threshold was 47.27 with a variance of 2.02. Analysis of this data set using similar conditions with *fourSig* (i.e. 10 separate runs on Chromosome 5, each with1000 permutations) resulted in significance thresholds of either 47 or 48 reads per window. While the average threshold was still 47.27, the variance was reduced by almost 10-fold to 0.22-fold (Figure 1C). In general, both methods resulted in similar thresholds on average, but the variance was always higher using the method described by Splinter *et al.* (2011). One possible explanation for the increased variance in the previous method is that the FDR calculation is dependent on averages, which can be highly influenced by extreme outliers. In practical terms, this increased variance may lead to skewed threshold calculations when the method is applied only once to a 4C-Seq data set. In contrast, the FDR calculations in *fourSig* are made after each randomization, and a single outlier cannot have a disproportionate effect on the other calculations. Based on this difference, *fourSig* should be more robust to outliers generated during read randomization, thus reducing the potential for misestimating a suitable significance threshold.

#### Background masking and prioritization features

*fourSig* uses two notable features to deal with common technical issues with interpretation of chromosome conformation data. 3C-based experiments typically find increased local contact probability associated with interacting regions and a disproportionate number of reads detected near the viewpoint. Therefore, it is possible that a single threshold calculated from the observed distribution of an entire chromosome may be set too high to detect long-range interactions. This tendency would lead to more conservative significance calls, as the threshold for the entire chromosome is weighted higher by the influence of viewpoint-proximal detections; however, it is reasonable to desire the implementation of variable position-dependent thresholds. To this end, *fourSig* allows the user to mask the reads for any specific region before determining a significance threshold. This feature will completely remove the masked area from analysis and is especially useful for the region surrounding the viewpoint because reads mapping to closely neighboring fragments can sometimes be orders of magnitude larger than those observed at more distal locations. Using this feature, one can mask out as large or as small an area as desired, thereby allowing for limitations of viewpoint-proximal background influence on threshold calculation.

Another important feature of *fourSig* is that it assigns a priority classification for interactions, based on the distribution of reads within the windows. When an interaction is captured between two loci with significant frequency, we expect that there will be a large number of reads for the fragment harboring the point of interaction (Figure 2A). Owing to various random events, such as incomplete digestions and increased local proximity, reads mapping to neighboring fragments should also be detected at a lower level. In the event of a consistent and frequent interaction, this behavior should give rise to a broader distribution of reads in the window because more opportunities for capture are available at the time of fixation. Conversely, this distribution of reads would not be expected at interactions detected owing to random chance.

Taking these expectations into consideration, we add additional criteria to the results after assessing the significance of a given window. For any given window, *fourSig* manipulates the 3C fragment with the most reads mapped to it and re-evaluates whether the window still passes the determined significance threshold (Figure 2B). We describe a window as being ‘Broad’ (Category 1) if the fragment containing the highest number of reads can be removed and the remaining read count from all other fragments still exceeds the significance threshold. Just as fragments surrounding the viewpoint will have many reads mapped to them because of their proximity, fragments surrounding a true interaction will also be in physical proximity to the viewpoint and have elevated probabilities of contact. Therefore, these types of windows reflect the expected fiber-like nature of chromatin and are most likely to be reproduced (see results below). If this test fails, the value for the fragment with the highest read count is reduced to the average value of the two adjacent fragments. If the window can still exceed the significance threshold, we designate it as ‘Intermediate’ (Category 2). Finally, if the window is only significant with the observed read counts, then it is designated as ‘Narrow’ (Category 3). Interactions that contain multiple windows are categorized based on the broadest designation assigned.

### Comparison of *fourSig* with existing 4C-Seq analysis methods

#### Verification of known Hbb-b1 interactions

The mouse beta-globin (*Hbb-b1*) locus contains several well-characterized long-range interactions that associate with gene expression states (27–29). Soler *et al.* (26) report 4C-Seq interaction data from *Hbb-b1* in two cell types: fetal liver (FL) cells, where beta-globin genes are active, and fetal brain (FB) cells, where the beta-globin genes are silent. One high-resolution 4C-Seq analysis method, r3CSeq (13), used these data as a benchmark for demonstrating sensitivity and accuracy. Additionally, an alternative method, 4Cseqpipe (12), generated an additional data set at this locus using a non-standard 4C-Seq protocol as a benchmarking experiment. To compare the precision and accuracy of *fourSig* with r3CSeq and 4Cseqpipe, we analyzed an *Hbb-b1* 4C-Seq data set with *fourSig* (26). These data, which have already been aligned, were downloaded from the r3cseq Web site: http://r3cseq.genereg.net/Site/index.html. We then converted the aligned reads to read counts per fragment with samToReTab.pl, a program included in *fourSig* (Supplementary Methods). Using equivalent settings (window size of 1 and only surrounding the Hbb1-b1 viewpoint), *fourSig* produced results that were nearly identical to those obtained with r3Cseq (Supplementary Figure S3A and B). Specifically, both methods detect interactions upstream of the viewpoint and at the beta-globin locus control region (LCR) in FL tissue when compared with FB tissue. Additionally, *fourSig* results are consistent with those obtained from 4Cseqpipe (15). Together, our results suggest that *fourSig* is as accurate as existing methods.

Owing to the way in which prioritization is determined, all enrichments determined by *fourSig* with a window size of one fragment are designated as Narrow interactions. Therefore, we chose to expand the window size and look chromosome-wide to see how interactions are categorized when more fragments are available for use. When the window size is expanded to five fragments, our method found that compared with the rest of the chromosome, the density of Broad interactions was higher with increasing proximity to the viewpoint (Supplementary Figure S3C and D, data not shown). Comparatively, there were approximately three times as many Broad interactions in FL cells than were observed in the FB tissue (60 and 18, respectively). As will be shown later, these results suggest a higher probability of reproducing the interactions near the viewpoint in the FL cells than in FB, which is consistent with LCR interactions facilitating expression of *Hbb-b1* in the FL. Furthermore, these results are consistent with previous claims that larger window sizes improve reproducibility (16).

#### Additional validation of fourSig against a Nanog 4C data set

In addition to *Hbb-b1* 4C, we validated our method against a second published 4C data set derived from mouse embryonic stem (ES) cells (30). The original experiment identified genome-wide interactions from the *Nanog* locus. *Nanog,* a transcription factor (TF) required for ES cell self-renewal, was found to be engaged in interactions with *Rybp*, *Tcf3*, *Ezh2* and *Smarcad1*, all genes implicated in the maintenance of ES cell pluripotency. Additionally, their results showed strong signals at the *Bcat1* and *Kras* loci. The statistical analysis by de Wit *et al.* (2013) was performed using a window size of 100 3C fragments; however, a much smaller window (W = 31) was used for visualization in their paper. Because the incorporation of read data in *fourSig* allows for relatively small window sizes, we performed an analysis using W = 31. When masking out a 50-kb region on both sides of the *Nanog* promoter, we also identified interactions with the *Rybp*, *Tcf3*, *Smarcad1*, *Bcat1* and *Kras* loci. In addition, these loci were assigned Broad priority classifications, indicating that their significance was not derived from a single fragment within a window (Supplementary Figure S4A). Interestingly, we were unable to detect significant interactions between *Nanog* and the *Rybp*, *Tcf3*, *Bcat1* and *Kras* loci with the r3Cseq software (data not shown).

The peak at *Ezh2* was relatively small compared with the other peaks (Supplementary Figure S4A), so we used *fourSig* to perform a localized analysis, similar to the approach used by de Wit *et al.* (2013). To approximate the ∼3000 HindIII fragment background model used in the previous study, we generated threshold values from a local distribution derived from the observed data within 4.5 Mb of either side of the *Ezh2* locus. This localized analysis identified a Broad significant interaction between *Nanog* and *Ezh2* (Supplementary Figure S4B).

Finally, we used the *Nanog* 4C data set to characterize the effects of different window sizes on the identification and categorization of interactions by *fourSig*. Using window sizes of 31, 10 and 5 HindIII fragments, we identified an inverse correlation between window size and number of detected interactions (Supplementary Figure S4C). However, the proportion of Narrow categorizations as a percentage of total interactions also increases as the window size is reduced (Supplementary Figure S4D). Therefore, while a reduction in window size may lead to a greater number of called interactions, the prioritization scheme is effective at illuminating which interactions are representative of single-fragment interactions.

#### Analysis of a gene with allele-specific expression bias

Long-range contacts have frequently been reported to have functional association to expression states (8,31,32). Therefore, we hypothesized that a gene whose alleles are differentially regulated may show divergent interaction profiles. To demonstrate the efficacy of *fourSig*, we designed and performed an allele-specific 4C-Seq experiment at the TSS of a gene that exhibits a strong allelic expression bias.

**Figure 3.**
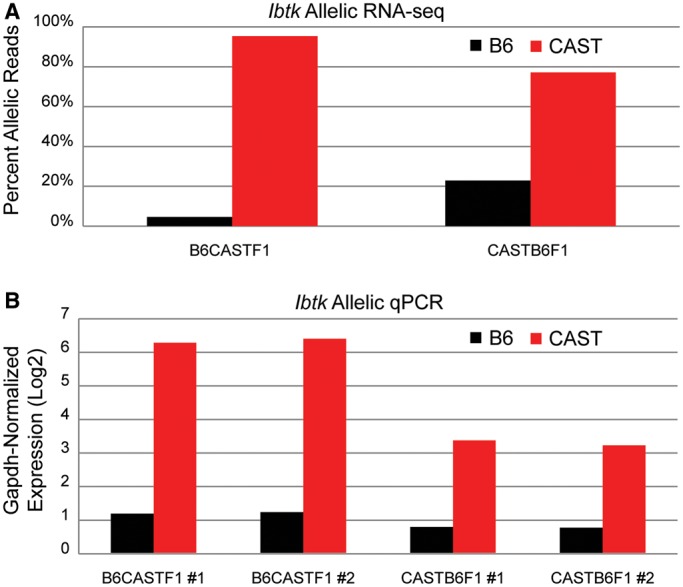
Validation of allelic expression bias in *Ibtk*. (**A**) The proportion of allele-specific reads detected from RNA-Seq experiments in two different TS cell lines shows a heavy expression bias in favor of the CAST allele for *Ibtk*. Cell lines are labeled to reflect the mode of parental inheritance of strain (maternal × paternal). (**B**) Expression bias of *Ibtk* was confirmed by allele-specific qRT-PCR. Each cell line was assayed twice, in triplicate. Results are displayed as Log2 transformations of expression relative to *Gapdh*.

Our laboratory previously produced allele-specific RNA-Seq data in TS cells derived from reciprocal F1 crosses between B6 mice and CAST mice (18). We searched these data for candidates that exhibited significant allelic expression bias in excess of 2-fold in favor of one allele. Because strain-specific differences in promoter-proximal TF binding sites, rather than allele-specific interactions with regulatory elements, could influence expression bias, we initially looked to exclude genes harboring sequence variations near the promoter (33). Using the JASPAR database (34), we extracted binding motifs for TFs expressed in TS cells and searched for binding sites that may be disrupted by sequence variations within 10 kb of the TSS for any biased genes. Most candidates contained comparable TF binding motifs (not shown), ruling out variations in known TF binding sites within promoter-proximal sequence as a likely source of regulation. We further constrained our candidate list by requiring that the 3C fragment containing the TSS also contained appropriately positioned restriction sites and SNPs for performing allele-specific 4C (Supplementary Figure S2). Ultimately, we settled on *Ibtk*, a gene encoding an inhibitor of Bruton’s tyrosine kinase (35,36), as a candidate for performing allele-specific 4C-Seq. The RNA-Seq data demonstrate that *Ibtk* expression is heavily skewed in favor of the CAST allele (Figure 3A). These results were verified using allele-specific qRT-PCR in multiple TS cell lines, both CASTB6F1 and B6CASTF1 (Figure 3B and Supplementary Figure S5).

#### 4C for Ibtk locus

Having confirmed the allelic expression bias for *Ibtk*, we designed a 4C-Seq strategy to identify allele-specific long-range interaction patterns (Supplementary Figure S6 and Supplementary Table S1). Three sequencing data sets representing two biological replicates were obtained and analyzed by *fourSig*. Each read is a hybrid of known and unknown sequences. The known sequence was analyzed for the presence of the B6 or CAST SNP to determine the allelic origin of the viewpoint (Supplementary Figure S6). Additionally, the unknown sequence was also analyzed for the presence of a known SNP to determine the allele to which the interaction occurred. Before analysis with *fourSig*, mapped reads were sorted into separate data sets depending on the allelic origin of the viewpoint and the allelic origin of the captured sequence.

Because *fourSig* uses read counts to determine significance threshold, it was imperative for us to rule out any interaction called due to technical errors such as PCR amplification bias. In our assay, this distinction can be made due to the inclusion of random barcodes incorporated between sequencing adapter sequences and 4C genomic primer sequences (Supplementary Figure S2). Repeated amplification of single contacts will generate reads containing identical barcodes on both ends of the read, creating an indicator of the degree to which amplification bias may be affecting the detection of a mapped 3C fragment. We inspected the barcodes in each of our data sets after sorting the mapped reads by allelic origin and found no fragments containing a disproportionate number of reads with identical barcodes (data not shown).

We hypothesized that allele-specific contacts may harbor putative distal regulatory elements. Therefore, window sizes were kept small to achieve high-resolution results. We analyzed *Ibtk* 4C-Seq data with *fourSig* using a window size of five 3C fragments and an FDR < 0.01. Using informative SNPs in the contacted regions, interactions were assigned to specific alleles (see Supplementary Methods). For the present analysis, we excluded *trans*-interactions mapping to the opposing Chromosome 9 of the analyzed viewpoint (Supplementary Figure S7). Interactions mapping to Chromosome 9 that lacked informative SNPs were assumed to be in *cis*. Extrapolation of the average proportion of *trans* calls in each library would suggest that <1% of the interactions may be erroneously called *cis*, making this a reasonably safe assumption.

**Figure 4.**
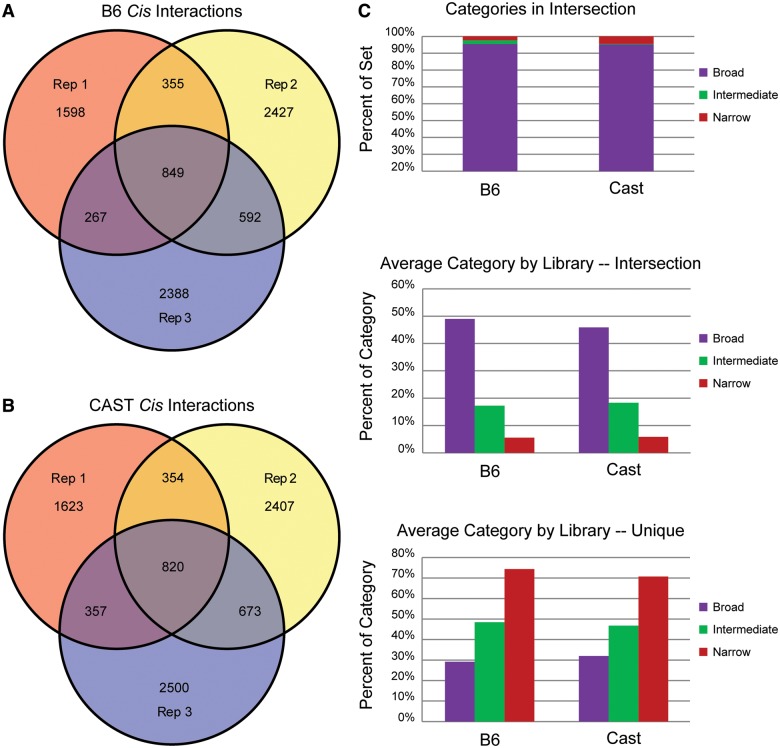
Significant interactions with the *Ibtk* TSS. (**A** and **B**) 3C fragments found in interactions have similar proportions of overlap between replicate 4C-Seq experiments. A similar number of interacting fragments were found in the intersection of replicates for both the B6 (A) and CAST (B) alleles. (**C**) Peak priority classifications for different sets of interacting fragments. Priority categories for replicated interacting fragments are shown as a proportion of the total intersection (top). Proportion of interactions assigned a classification in each replicate are averaged for B6 and CAST alleles for fragments found in the replicate intersection (middle) and fragments unique to a replicate (bottom).

To focus analysis on the locations of precise overlap between replicates, individual 3C fragments found within the *cis*-interactions were compared among the three replicates for each viewpoint allele. The intersection of these libraries is defined as the set of consistent interacting fragments from interactions detected for each viewpoint (Figure 4A and B). The utility of the peak prioritization algorithm can be demonstrated through the distribution of category calls among the replicate sets. The overwhelming majority of the fragments that were consistently detected among replicates tend to belong to Broad interactions (Figure 4C, top). To understand how informative the classification scheme may be with respect to a single replicate, the proportions of interaction classifications relative to all interactions in single replicates were compared. On average, a substantially larger portion of fragments belonging to Broad interactions within individual replicates were present in the intersection of all three replicates (Figure 4C, middle). Conversely, the majority fragments belonging to interactions classified as Narrow tended to be unique to that particular replicate (Figure 4C, bottom). These data demonstrate the classification scheme offered may predict the likelihood that an interaction will be consistently identified among many replicates.

**Figure 5.**
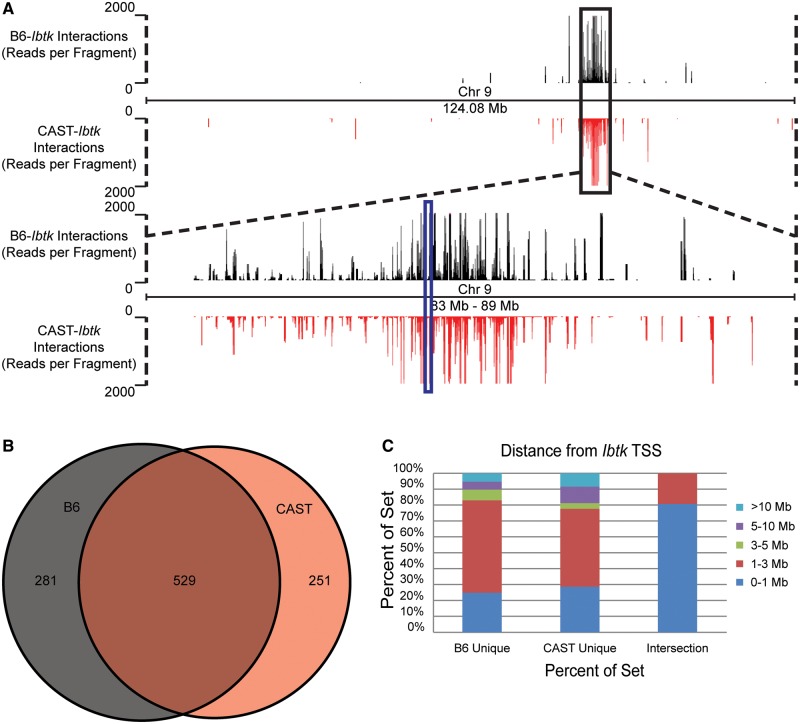
Replicated *cis*-interactions for the *Ibtk* TSS. A Distributions of detected reads (black = B6 and red = CAST) for replicated, Broad interacting fragments were plotted along Chromosome 9 (upper panel). The region surrounding the *Ibtk* locus (blue box) is expanded (lower panel). (**B**) Intersection of Broad *cis*-interactions for the entire chromosome between the B6 and CAST alleles. (**C**) A proportional breakdown of the allelically unique and common interaction sets by distance from the *Ibtk* TSS.

To ensure that further analysis focused on high-confidence contacts, we selected only fragments from Broad interactions that were represented in all three 4C-Seq data sets. To visualize the results from the experiments, reads from each data set were summed per 3C fragment and plotted along Chromosome 9 (Figure 5A). The majority of reads from repeated interactions were located within 3 Mb of the viewpoint (Figure 5A, expanded region). Additionally, we found that the majority of reads within these contacts were shared between alleles (∼66%, Figure 5B), especially in the region upstream of the *Ibtk* TSS (Figure 5A). As the distance from the viewpoint increased, allele-specific differences in the location of contacts become more apparent. In fact, at a distance of 3 Mb, interactions approach near-allelic exclusivity (Figure 5A). Analysis of the positioning of interactions relative to the viewpoint validates this visual observation (Figure 5C). No interacting fragments found in common between alleles were found further than 3 Mb from the viewpoint. However, ∼80% of the interacting fragments found exclusively on one allele were also located within 3 Mb of the viewpoint. Therefore, despite sharing a largely similar spatial organization, there were a substantial number of allele-specific contacts that are associated with one allele versus another.

#### Validation of allele-specific contacts by FISH

**Figure 6.**
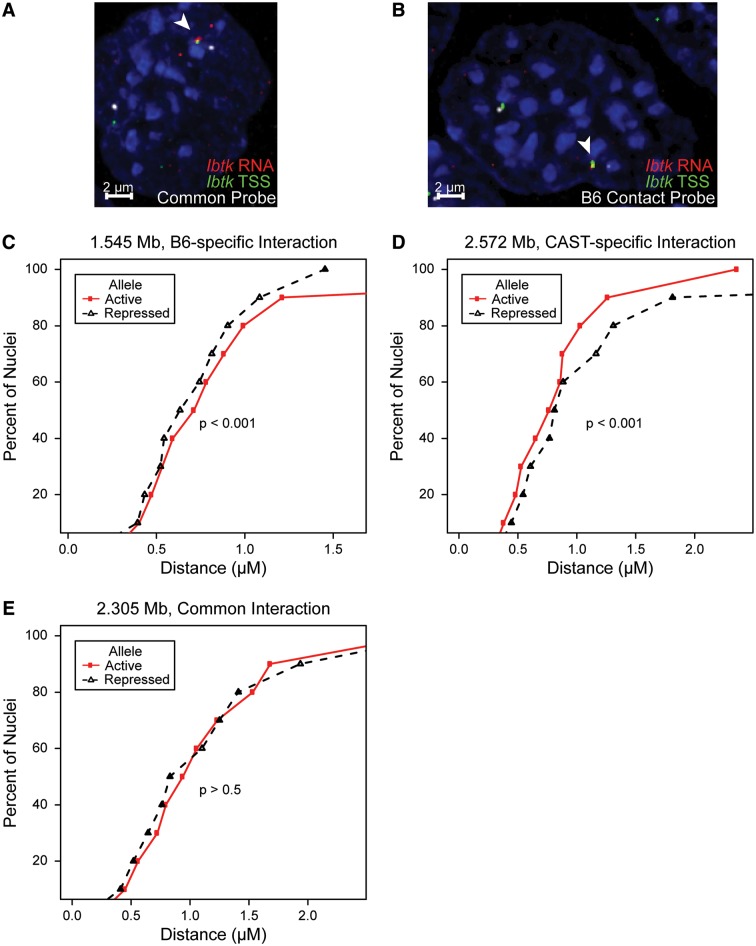
Validation of selected interactions by FISH. (**A** and **B**) Representative FISH images of a common interaction (A) and a B6-specific interaction (B) are displayed. Active allele (arrowhead) is determined by colocalization of RNA signal (red) with the *Ibtk* TSS (green). Distance is measured from the centers of the *Ibtk* signal and the signal from the interacting probe (white). Nucleus is counterstained with 4',6-diamidino-2-phenylindole (blue). (**C–E**) The percentage of scored nuclei is plotted against the distance between the probed locus and the *Ibtk* TSS as measured by DNA-FISH. The measured loci represent interactions identified by 4C to be specific to the B6 allele (C, *n* = 56), specific to the CAST allele (D, *n* = 51) or common to both alleles (E, *n* = 51). The expression state of *Ibtk* was assessed by colocalization of a RNA signal with the *Ibtk* TSS. Measurements of active alleles are traced in red, while repressed alleles are traced in black. All distances are in micrometers. Significance of trends was determined using an exact binomial test.

To test the validity and allelic nature of the interactions identified by the 4C experiment, we selected three interactions to test by FISH. DNA-FISH probes were made to measure two allele-specific *cis*-interactions, one for B6 and one for CAST, and one interaction common to both alleles (Supplementary Figure S8A). Because the majority of allele-specific contacts were found within 1–3 Mb of the TSS, interactions for verification were chosen from loci within this space. To ensure that the FISH test was reflective of the high resolution and precision with which *fourSig* calls interactions, loci for validation of allele-specific contacts were selected so that the called interactions were not isolated by large linear distances from interactions found on the opposing allele. Because SNP differences cannot be used to discriminate between alleles in FISH experiments, we used the allelic bias in *Ibtk* expression to identify the B6 or CAST allele. As such, the colocalization of *Ibtk* expression by RNA-FISH with a DNA-FISH probe for the *Ibtk* TSS was used to identify the active (CAST) allele (Supplementary Figure S8B). We observed monoallelic expression in 92.8% of scored nuclei, confirming that the use of RNA-FISH is an acceptable indicator for allelic discrimination (data not shown). For each experiment, DNA-FISH probes for the *Ibtk* TSS and the assayed interaction were cohybridized with the *Ibtk* cDNA probe. Measurements were taken from the centers of DNA signals (Figure 6A and B). For the allele-specific interactions, the measured distance between *Ibtk* and the interacting locus was consistently smaller for the expected allele (*P* < 0.001). The repressed allele was consistently closer in space to the B6-specific interaction, whereas the opposite was found for the CAST-specific interaction (Figure 6C and D, respectively). No discernible difference in the measure distances between loci was detected for the interaction common to both alleles (*P* > 0.5, Figure 6E). These results validate the interactions identified by the 4C-Seq experiments and *fourSig* analysis.

#### Potential regulatory differences associated with differential interactions

**Figure 7.**
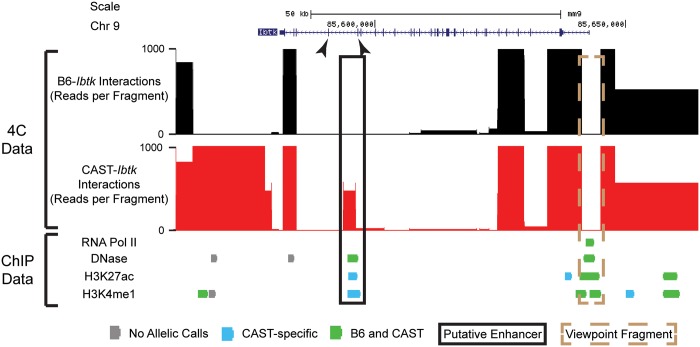
Allele-specific interaction at a putative enhancer for *Ibtk*. A UCSC Genome Browser screenshot of 4C interaction data and selected chromatin data at the *Ibtk* locus are shown. Exons 24 and 25 of *Ibtk* are highlighted with arrows. The 3C fragment containing the *Ibtk* TSS is indicated by a dashed brown rectangle. A putative intragenic enhancer aligning with a previously reported regulatory element is enclosed by a solid black rectangle. For chromatin immunoprecipitation sequencing tracks, green boxes indicate biallelic enrichment, blue boxes indicate CAST-specific enrichment and gray boxes indicate insufficient SNP detection to make an allele-specific call.

Closer visual analysis of the allelic interaction data revealed that, in some instances, strong differences in read counts can indicate a preferential allelic contact even when the 3C fragment in question is found in interactions called for both alleles. Notably, we identified a 3C fragment containing more reads interacting with the CAST allele versus the B6 allele (Figure 7). We examined this fragment for a combination of genomic features associated with active enhancer elements, namely, H3K27ac, H3K4me1 and DNase (37–39), previously assessed in TS cells (18). Our analysis demonstrates that the CAST 3C fragment is enriched for these marks relative to the B6 fragment. While this fragment is located only ∼50 kb downstream of the TSS for *Ibtk*, the difference in read counts between the alleles is striking. Also of interest is that a previous study of the human *Ibtk* locus identified the existence of a TSS for an alternative transcript for *Ibtk* located in approximately the same relative location (36). This raises the possibility that in addition to acting as a TSS for an alternative transcript, modification of this site may act as an enhancer whose contact with the promoter is necessary for activating transcription of the gene. The precedent of this locus for having regulatory function combined with the presence of active histone marks and the detection of allele-specificity in our 4C-Seq analysis lends credibility to the ability of *fourSig* to identify interactions with putative distal regulatory elements.

#### Allelic Ibtk 4C analysis using r3Cseq

To determine whether the application of *fourSig* is advantageous in utility over an existing method, a similar analysis was performed using r3Cseq (16) with default parameters to determine significant interactions. In comparison with the *fourSig* analysis, few interactions were determined in each individual replicate, leading to near 40- and 30-fold reductions in replicated interacting fragments for B6 and CAST, respectively (Supplementary Figure S9A and B). Of the fragments from interactions found in all replicates, none were detected by r3Cseq that were not also found in the *fourSig* analysis (Supplementary Figure S9C and D). Additionally, although the small sample size skews the exact relative proportions, we did find approximately similar proportions of overlaps between allelic interactions as determined by r3Cseq (i.e. 50 and 75% common interactions for B6 and CAST, respectively) (Supplementary Figure S9E).

An inspection of the positioning of consistent interactions from each allele found that, similar to what was observed in the *Nanog* analysis, *r3Cseq* detected consistent interactions only proximal to the viewpoint for both alleles (Supplementary Figure S10). Although no interactions were detected further than 1.1 Mb from the viewpoint, the trend of greater dissimilarity in allelic interactions further from the viewpoint seen earlier was preserved (Supplementary Figure S10A). Notably, all of the interactions detected by *fourSig* and validated by FISH were beyond the range of the most distal interactions found by *r3Cseq*. Finally, the *r3Cseq* method failed to consistently detect contact with putative enhancer described in Figure 7 in either allele (Supplementary Figure S10B). Therefore, this comparison demonstrates that despite a substantially simpler method of detecting significant interactions, *fourSig* is more sensitive to the detection of validated interactions occurring at further distances from the viewpoint and highly probable allelic interactions with regulatory elements that have published precedent for functional activity.

## DISCUSSION

In this study, we introduced a new method, *fourSig*, for analyzing 4C-Seq data. *fourSig* uses the full range of quantitative information derived from read depth, produces consistent results and can inform as to the reproducibility of interactions among replicate experiments.

Similar to the recently published methods *r3Cseq* (16) and *4Cseqpipe* (15), *fourSig* uses the full range of quantitative information derived from read depth to determine significance thresholds for 4C-Seq data. Previous 4C-Seq analyses have typically discarded this information due to concerns over possible PCR amplification bias during library preparation (13,14). Instead, these studies use a transformation of the read counts to Boolean values, leading to 3C fragments being ruled simply as detected or not detected. This binarization of the data necessitates the use of window sizes of at least 100 fragments, which generates a lower-resolution analysis of the data. As a result, these methods would likely not be able to detect the specific LCR interactions within the beta-globin locus (26) or the putative regulatory element described here for *Ibtk*. The incorporation of read counts into threshold calculation allows the use of much smaller windows and improves the resolution available for 4C-Seq experiments. Therefore, higher-resolution analysis methods such as *fourSig*, *r3Cseq* and *4Cseqpipe* represent a substantial improvement over previous limitations.

There are two ways to determine the threshold for significance when using permuted data. *fourSig* calculates a cutoff for the FDR after each permutation and then picks an ultimate threshold from the distribution of cutoffs. Alternatively, as is used in the study by Splinter *et al.* (2011), it is possible to perform all of the permutations first and use the average of the cumulative data set to identify a single cutoff for the FDR. Initially, we used both methods, but found that the former method provides less variation among repeated analysis of data sets and is likely to be more robust to extreme values that occurred in rare permutations. Additionally, while the use of a single threshold for an entire chromosome may result in overly conservative significance calls, especially for interactions with loci at large distances, the use of the masking feature, as demonstrated for the *Nanog* to *Ezh2* interaction, is very effective for making refined assessments at any particular region of interest. This is a powerful feature in *fourSig*, which can allow the user to fine tune the sensitivity of interaction detection by tailor-suiting a background model to any location or locus size desired. Taken together, *fourSig* provides an easy-to-use method for high-resolution detection of genomic interactions while offering the user the flexibility to find optimal conditions for gaining informative results.

The interaction prioritization feature represents a novel addition to 4C-Seq analysis and a useful improvement over existing methods. In addition to the consistent application of a Broad designation to many well-described interactions from published data sets, the trends of increased reproducibility for Broad interactions demonstrated in replicate analysis of the *Ibtk* data lend strength to this feature’s role in flagging potentially interesting interactions.

However, while the interaction prioritization feature in *fourSig* offers predictive value in terms of the likelihood that detected interactions may be reproduced, it should be noted that interactions classified in all three categories are capable of being consistently reproduced. Our analysis of the intersection of replicates showed that >5% of the consistently detected 3C fragments were found within interactions designated as Narrow or Intermediate. This could occur, for example, in the event that the dominant 3C fragment in a window is large, leaving little linear distance to either side of the primary point of contact to capture neighboring fragments. However, this is likely to be an infrequent event when using a 6- or 4-cutter for generating the initial 3C library. Additionally, it is not our intention to suggest that the use of this feature may serve as a replacement for performing experimental replicates. As is evidenced by the analysis of the *Ibtk* data, a sizable portion of the Broad interactions are not identified in all of the replicates. Instead, a more appropriate use of this feature would be to serve as a guide for emphasizing interactions in the event that few replicates are possible or as a support to avoid seeking diminishing returns yielded from excessive replicate experiments.

One biological context in which the functional role of higher-order chromatin organization may have particular importance is in allelic expression bias. Differential expression patterns between alleles are traditionally associated with genomic imprinting (40,41); however, allelic biases unrelated to parent-of-origin effects have been reported to affect as much as 12% of transcripts in laboratory mouse strains (19). Allele-specific expression bias implies the existence of differential regulatory schemes between alleles of the same gene; schemes that may be potentially related to spatial organization. Although our results for *Ibtk* do not prove that functional regulatory elements lie within the described interactions, the consistency at which we detected allele-specific interactions supports the notion that functional association of conformation with differential expression state is a possible source of regulatory control.

Our results at the *Ibtk* locus indicate that the majority of interactions detected within 1 Mb of the viewpoint are shared between alleles. This is consistent with numerous 4C studies that report substantially increased contacts with loci at shorter linear distances from the viewpoint (2,14,42,43). Furthermore, our data are consistent with the existence of topologically associated domains, where *cis*-interactions tend to occur with highest probability in discrete windows of up to 1 Mb of linear distance (12,24,25). The association of a putative enhancer with an interaction containing more reads on the CAST allele demonstrates that keeping read counts, rather than reducing them to Boolean values, is useful for identifying allelic differences in conformation. In fact, the observation of a bias in relative detection and the proximity of this site to the *Ibtk* TSS underscore the importance of incorporating read counts into the analysis method.

In this study, we introduced a new method, *fourSig*, for analyzing 4C-Seq data. We showed that this method is capable of reproducing well-studied interactions identified by other methods and can be more sensitive in determining significant interactions between large linear distances than some existing methods. The combination of accessibility to high-resolution windowing, not possible with binarization of data, and sensitivity to detection of distal interactions, not demonstrated by *r3Cseq*, allows *fourSig* to deliver a sort of ‘best of both worlds’ result compared with currently available methods. Furthermore, it is of noteworthy significance that we were able to validate allele-specific interactions detected for *Ibtk*. Measurements between FISH signals are typically limited in resolution to ∼0.2 µm. Because the DNA in the nuclei exists as compacted chromatin, 1–3 Mb does not always lead to large physical distances in separation. Therefore, the limitation of resolution for FISH can make differential measurements between copies difficult owing to the natural proximity of loci within this range. That we were able to consistently verify proximity trends of detected interactions at this range by FISH speaks to the precision with which interactions can be called by *fourSig*. The validation of *fourSig* against existing and novel data and the comparative performance to existing algorithms demonstrate that the methods described here will be useful in assisting investigators with analysis of 4C-Seq data and detection of relative differences in contact probabilities.

## AVAILABILITY

All Perl and R scripts are available at SourceForge: http://sourceforge.net/projects/foursig/. A tutorial for their use may be found at: http://starmer.med.unc.edu/∼jstarmer/fourSig/TUTORIAL.html. 4C-Seq data for the beta-globin locus were downloaded at: http://r3cseq.genereg.net/.

## ACCESSION NUMBERS

The NCBI accession number for the *Nanog* 4C-Seq data (30) is GSE37275. The NCBI accession number for the TS cell data used to determine allelically biased candidates (18) is GSE39406. The NCBI accession number for the raw 4C-Seq data for *Ibtk* is GSE50907.

## SUPPLEMENTARY DATA

Supplementary Data are available at NAR Online.

## FUNDING

National Institutes of Health (NIH) [R01GM10974 to T.M., NIH F32-CA144389 to J.W.M.]. Funding for open access charge: NIH.

*Conflict of interest statement*. None declared.

## Supplementary Material

Supplementary Data
