# Supplementary material for: fourSig: a method for determining chromosomal interactions in 4C-Seq data
Source: Nucleic Acids Res. 2014 Feb 20;42(8):e68. doi: 10.1093/nar/gku156 (PMC4005674; doi:10.1093/nar/gku156)
Supplement: Supplementary Data [file supp_42_8_e68__index.html]

fourSig: a method for determining chromosomal interactions in 4C-Seq data — fourSig: a method for determining chromosomal interactions in 4C-Seq data — Supplementary Data 

# *fourSig*: a method for determining chromosomal interactions in 4C-Seq data

## Supplementary Data

files

**Files in this Data Supplement:**

- Supplementary Data - pdf file
