# Supplementary material for: fourSig: a method for determining chromosomal interactions in 4C-Seq data
Source: Nucleic Acids Res. 2014 Feb 20;42(8):e68. doi: 10.1093/nar/gku156 (PMC4005674; doi:10.1093/nar/gku156)
Supplement: Supplementary Data [file supp_gku156_nar-03660-met-n-2013-File009.pdf]

fourSig: A Method for Determining Significantly Enriched Interactions in 4C-Seq Data

Rex L. Williams, Jr, Joshua Starmer, Joshua W. Mugford, J. Mauro Calabrese, Piotr Mieczkowski, Della Yee and Terry Magnuson*

Department of Genetics, The University of North Carolina at Chapel Hill, Chapel Hill, NC, 27599, USA
Carolina Center for Genome Sciences, The University of North Carolina at Chapel Hill, Chapel Hill, NC, 27599, USA
Lineberger Comprehensive Cancer Center, The University of North Carolina at Chapel Hill, Chapel Hill, NC, 27599, USA

* To whom correspondence should be addressed. Tel: +1-919-843-6475; Fax: 919-843-6365 Email: trm4@med.unc.edu

The authors wish it to be known that, in their opinion, the first 3 authors should be regarded as joint First Authors

SUPPLEMENTARY MATERIALS

TABLE OF CONTENTS

SUPPLEMENTARY METHODS 3

Allele-Specific qRT-PCR Assay for *Ibtk* 3

Sequence Alignment 3

Preparation of Data for Analysis with *fourSig* 4

FISH Methods 5

SUPPLEMENTARY TABLES 7

SUPPLEMENTARY FIGURES 9

REFERENCES 18

SUPPLEMENTARY METHODS

Allele-Specific qRT-PCR Assay for *Ibtk*

An intron-skipping amplicon was designed to detect allele-specific *Ibtk* transcripts by using a forward primer in which the most 3' nucleotide is a known SNP between B6 and CAST (Table S1) and a common reverse primer. PCR efficiency for each amplicon was calculated using 10-fold serial dilutions of cDNA derived from homozygous B6 or CAST TS cells. Allele-specificity for each forward primer was verified by performing qPCR where the forward primer and cDNA library were isotype matched and where primer and cDNA isotypes were mismatched (Figure S3). We did not observe amplification of any PCR product when primers and isotypes were mismatched. qPCR verification of *Ibtk* allelic expression bias was then performed in triplicate for at least two separate cDNA preps from all F1 TS cell lines. Average ∆C(t)s were normalized against *Gapdh* expression using the Pfaffl method for generating reference-normalized ratios for qPCR data (1).

Sequence Alignment

We used the mm9 genome assembly of *M. m. musculus* for the B6 genome (2). We generated a CAST genome sequence by replacing the appropriate nucleotides in mm9 with reported SNPs for *M. m. castaneus* (3). For *Ibtk* 4C data, raw sequence was handled using custom Perl scripts to identify the expected known sequence from the *Ibtk* viewpoint and to identify the appropriate allele based on the expected SNP in the fragment. In order to use the data for further analysis, we required that a roughly equal proportion of reads be obtained from each allele, validating that one copy was not preferentially amplified and detected (data not shown). After identification of the source allele and separation of the known sequence from the unknown captured sequences, the unknown portion of the reads were mapped to both the B6 and CAST genomes using the Bowtie algorithm (version 0.12.7) (4). We allowed for 2 mismatches and retained only sequences that mapped uniquely to the genome (settings –n 2 –l 100 –m 1 –best –strata). Successfully mapped reads were then paired, where possible, to increase the likelihood of detecting an informative SNP in the captured sequence. Reads were discarded in the event that the forward and reverse reads mapped to different regions of the genome.

Mapped reads were searched for SNPs by using the positions of known SNPs between B6 and CAST and ensuring that mismatches did not overlap with a SNP. Because captured sequence does not always contain a SNP, mapped reads files were generated for only data containing SNP information in the captured sequence ("allelic") as well as for all data regardless of the presence of a SNP ("non-allelic"). "non-allelic" reads files were then adjusted by removing known allelic reads that are inappropriate for the specific mapped reads file (i.e., reads aligned to CAST are removed from "non-allelic" files aligned to B6. This results in 4 "allelic" and "non-allelic" mapped reads files for each allele (B6 to B6, B6 to CAST, CAST to B6, and CAST to CAST). Since allelic information for the viewpoint is always retained, the adjusted "non-allelic" mapped reads files are used to generate input files for *fourSig* to maximize data available for analysis of *cis* interactions.

For *Nanog* 4C data, known viewpoint sequence was removed from each read using custom Perl scripts and the remaining captured sequence was aligned using the Bowtie algorithm. No mismatches were allowed because the captured sequence was relatively short and only uniquely mapped sequences were kept (settings –n 1 –m 1 –best). The mm9 genome assembly of *M. m. musculus* was used as the reference for this alignment.

Preparation of Data for Analysis with *fourSig*

The *fourSig* suite is primarily intended to provide support for identifying and prioritizing significant interactions in 4C-Seq data. It can work with data derived from a variety of 4C-seq protocols, including those that use different numbers of restriction enzymes to reduce fragment sizes or different sequencing strategies, such as paired-end or single-end. However, alignment of the sequence data is the responsibility of the user and should be suited to the specifics of the experimental design. Briefly, our handling of *Ibtk* sequence data focused on separating unknown sequence from viewpoint sequence in each read, using Bowtie to map the unknown portions to full B6 and CAST genomes, and identifying mismatches that suggest probable SNPs in the viewpoint portions (see "Sequence Alignment for *Ibtk* 4C Data"). Alternatively, one could generate a fragmented genome that only contained sequences where alignments were possible.

The steps described for aligning the sequence data and formatting the data for analysis by *fourSig* assumes that 4C libraries were generated from a dual restriction strategy, as demonstrated for *Ibtk* (Figure S2 and S6), and detected by paired-end sequencing. Depending on the needs of the investigator, 4C-Seq experiments may be designed using different fragmentation and sequencing strategies. In order to use the *fourSig* analysis tools, the raw data must be aligned and arranged into the TAB format that is used as an input file for the *fourSig* functions (Figure S1).

The TAB format is a tab-delimited file describing the observed alignment of sequence data and mappability of experiment specific restriction enzymes. The fields consist of chromosome location ("chr"), position ("pos"), number of reads ("reads"), indication of mappability ("map"), presence of a secondary restriction site within primary restriction fragment ("4bp"), indication that a fragment will be too short to detect ("short"), indication of whether a linearizing enzyme will disrupt mappability ("6bp interferes"), and the size of the sequence available for detection within the fragment ("unknown length") (column headers are in parentheses). To facilitate the generation of these files, two Perl programs (bowtieToReTab.pl and samToReTab.pl) are included with the *fourSig* suite at http://sourceforge.net/projects/foursig/. When supplied with some experiment specific information (see tutorial at http://starmer.med.unc.edu/~jstarmer/fourSig/TUTORIAL.html) and appropriate restriction site coordinate files, these programs will generate TAB files from any aligned sequence data in either the Bowtie output format (4) or the SAM format (5). A few common restriction site coordinate files are included in the *fourSig* suite as an example and others can be made as needed by the user from simply listing the coordinates from the desired reference genome in an identical format. For example, if one used additional restriction enzymes to generate smaller 4C fragments, the restriction site coordinate file would need to contain the locations of the cut sites for all enzymes. All provided files were made using the mm9 genome assembly.

If a 4C library is generated using a different strategy (i.e., not the double restriction strategy described for *Ibtk*), the *fourSig* program may still be used for analysis. At a minimum, *fourSig* only requires the first three fields (“chr”, “pos”, “reads”) for analysis. The mappability column is used in the event that the user wishes to exclude unmappable fragments from the threshold calculation, while the remaining fields clarify why a fragment is or is not mappable. A makeshift TAB file can be generated for 4C data from any type of preparation by simply providing the information necessary in the first three fields and by setting the “only.mappable” parameter to FALSE in the command invocations (see http://starmer.med.unc.edu/~jstarmer/fourSig/TUTORIAL.html).

Since *fourSig* uses non-binarized data in its analysis, it is advisable that precautions be taken to reduce the potential for amplification bias. If a barcoded strategy is used in the amplification of 4C templates, as is described for *Ibtk* (Figure S2), analysis of the barcodes in the aligned sequence data may be used to identify sources of possible amplification bias. Two programs, PCRDupCheck.pl and associateAndCheckBarCodes.pl, are included in the *fourSig* suite to perform this analysis. Using the FASTQ files, size of the barcode, and the Bowtie alignment outputs, PCRDupCheck.pl will generate of list of sequence IDs for each barcode detected. Then, associateANDCheckBarCodes.pl uses this list, along with viewpoint coordinates, to identify captured fragments in which a sizable portion of the signal can be accounted for by a single barcode and reports the number of unique barcodes for mapping to the fragment. The number of mapped reads for such a fragment may be replaced with the number of unique barcodes to ensure that the *fourSig* analysis does not count a signal that may be contaminated by excessive amplification. For both of these files, more detail may be found by referring to the annotations and summaries available within the source code.

Once tab files have been generated, and adjusted for barcode duplication if desired, the data is ready to perform analysis using the *fourSig*.R program. Instructions on specifying user-defined parameters for *fourSig* analysis may be found at http://starmer.med.unc.edu/~jstarmer/fourSig/TUTORIAL.html.

FISH Methods

TS cells for FISH experiments were grown for one passage off of feeder cells on gelatin-coated coverslips and fixed with 2% paraformaldehyde for 10 minutes at room temperature. After fixation, cells were permeablized by rinsing with chilled 1X PBS for 30 seconds, 0.5% Triton X-100 in 1X PBS for 1 min, and again with 1X PBS. Coverslips intended for use as RNase controls were treated with 10mg/mL RNase A (Sigma-Aldrich) for 10 minutes at 37 ^{o}C. Coverslips were dehydrated with a gradient of increasing ethanol concentration and heat denatured at 80 ^{o}C in a 70% formamide/2X SSC solution for 10 minutes. The cDNA probe was initially hybridized at 37 ^{o}C for at least 4 hours, followed by hybridization with the desired fosmid probes at 37 ^{o}C overnight. Hybridized coverslips were washed with 3 changes of 50% formamide/2X SSC, followed by 3 washes with 1X SSC, and then 2 washes with 0.5X SSC. Each wash was for 5 minutes at 42 ^{o}C. DAPI (Molecular Probes) was added to the first wash solution to counterstain DNA. Washed coverslips were mounted with ProLong Gold Antifade (Molecular Probes) and allowed to cure overnight at room temperature.

Tests with and without RNase treatment were performed to ensure that the cDNA probe detected only transcribed product and not genomic DNA (data not shown). For each probed interaction, validation experiments were performed by simultaneous detection of Ibtk RNA, the Ibtk locus, and the intended interaction. In order to be scored for measurement, we required that a nucleus have clear boundaries in the DAPI stain, biallelic signal from both DNA probes, and a clear RNA signal that overlaps with the Ibtk locus (Figure 6A and B). At least 50 nuclei were scored and counted for each experiment.

SUPPLEMENTARY TABLES

Supplemental Table 1. Primer Sequences.

Name	Purpose	Sequence (5'->3')	Description
js_ibtk_1fb	Allelic-qPCR	CTGTGCGAGACTTGAGTATTTCC	Forward cDNA primer, B6-specific
js_ibtk_1fc	Allelic-qPCR	TCTGTGCGAGACTTGAGTATTTCT	Forward cDNA primer, CAST-specific
js_ibtk_1r	Allelic-qPCR	GCCACGACAAGAACACTACAA	Reverse cDNA primer
Gapdhf1	qPCR	TGTTCCTACCCCCAATGTGT	Forward primer
Gapdhr1	qPCR	TGTGAGGGAGATGCTCAGTG	Reverse primer
Gapdh-H16n1	3C Ligation Control	ATACCAGCGGAGGGTTTCTT	Primer at 3' of HindIII fragment for amplification test of ligation efficiency
Gapdh-n1	3C Ligation Control	ATTGTTCCACAGTACGCCAGT	Primer at 3' of HindIII fragment for amplification test of ligation efficiency
4C_Ibtk-Nla3_Nex	Ibtk-4C	[Adapter Sequence]-NNNNTTTCTGATCCTTGTTGAAGCAG	NlaIII side primer with random barcode and 3' end of Nextera V1 Universal Adapter for Ibtk-4C amplification
4C_Ibtk-H3_Tru	Ibtk-4C	[Adapter Sequence]-NNNNTCTGTGTTCCTTCCTAGTGTACTTTT	HindIII side primer with random barcode and 3' end of Illumina TruSeq Adapter for Ibtk-4C amplification
IbtkExonOligo_1F2	Ibtk RNA FISH Probe	GTGTTGGACTGGCTGATTGA	Forward primer in Exon 2
IbtkExonOligo_1R3	Ibtk RNA FISH Probe	TGAGTTGGCCTGTCCTTCAT	Reverse primer in Exon 3
IbtkExonOligo_2F1	Ibtk RNA FISH Probe	CGGAAGCCAGAATAGCAAAC	Forward primer in Exon 4
IbtkExonOligo_2R1	Ibtk RNA FISH Probe	CTGGGACAGAAACACCGAAT	Reverse primer in Exon 5
IbtkExonOligo_3F1	Ibtk RNA FISH Probe	CAGGCTTGTGGAAGGACTGT	Forward primer in Exon 6
IbtkExonOligo_3R2	Ibtk RNA FISH Probe	GAGTCCACAGCACCGTATGA	Reverse primer in Exon 7
IbtkExonOligo_4F3	Ibtk RNA FISH Probe	GGTTCTTGTCTCTGGAGGTTG	Forward primer in Exon 9
IbtkExonOligo_4R1	Ibtk RNA FISH Probe	ATTGACACCTGGCGAGGATA	Reverse primer in Exon 10
IbtkExonOligo_5F1	Ibtk RNA FISH Probe	AGTGTTCTTGTCGTGGCTGA	Forward primer in Exon 16
IbtkExonOligo_5R2	Ibtk RNA FISH Probe	TCGCAGCATTCTTAAGGGTAA	Reverse primer in Exon 17
IbtkExonOligo_8F2	Ibtk RNA FISH Probe	CAATTCAGGAACGAACAGCA	Forward primer in Exon 24
IbtkExonOligo_8R1	Ibtk RNA FISH Probe	GCAACATTTAGGGCTGGAGA	Reverse primer in Exon 25

Table S1. Primers Sequences. Primers used for qPCR, 3C and 4C library preparation, and RNA-FISH Probe are listed.

Supplemental Table 2. BAC and Fosmids.		
Clone	Location	Purpose
RP23-356F10	chr6:125044436-125275813	Generation of amplification controls for 3C ligation efficiency test
WI1-1164K11	chr9:85642568-85680931	DNA FISH Probe 1, *Ibtk* TSS
WI1-1905A07	chr9:87194252-87232416	DNA FISH Probe 2, B6-specific interaction with *Ibtk* TSS (Repressed allele)
WI1-1702H10	chr9:88221104-88263244	DNA FISH Probe 3, CAST-specific interaction with *Ibtk* TSS (Active allele)
WI1-0698D21	chr9:87954089-87992722	DNA FISH Probe 4, Interaction with *Ibtk* TSS common to both alleles

Table S2. BAC and Fosmids. Clones for 3C ligation efficiency control and DNA-FISH probes are listed along with their associated genomic locations.

SUPPLEMENTARY FIGURES

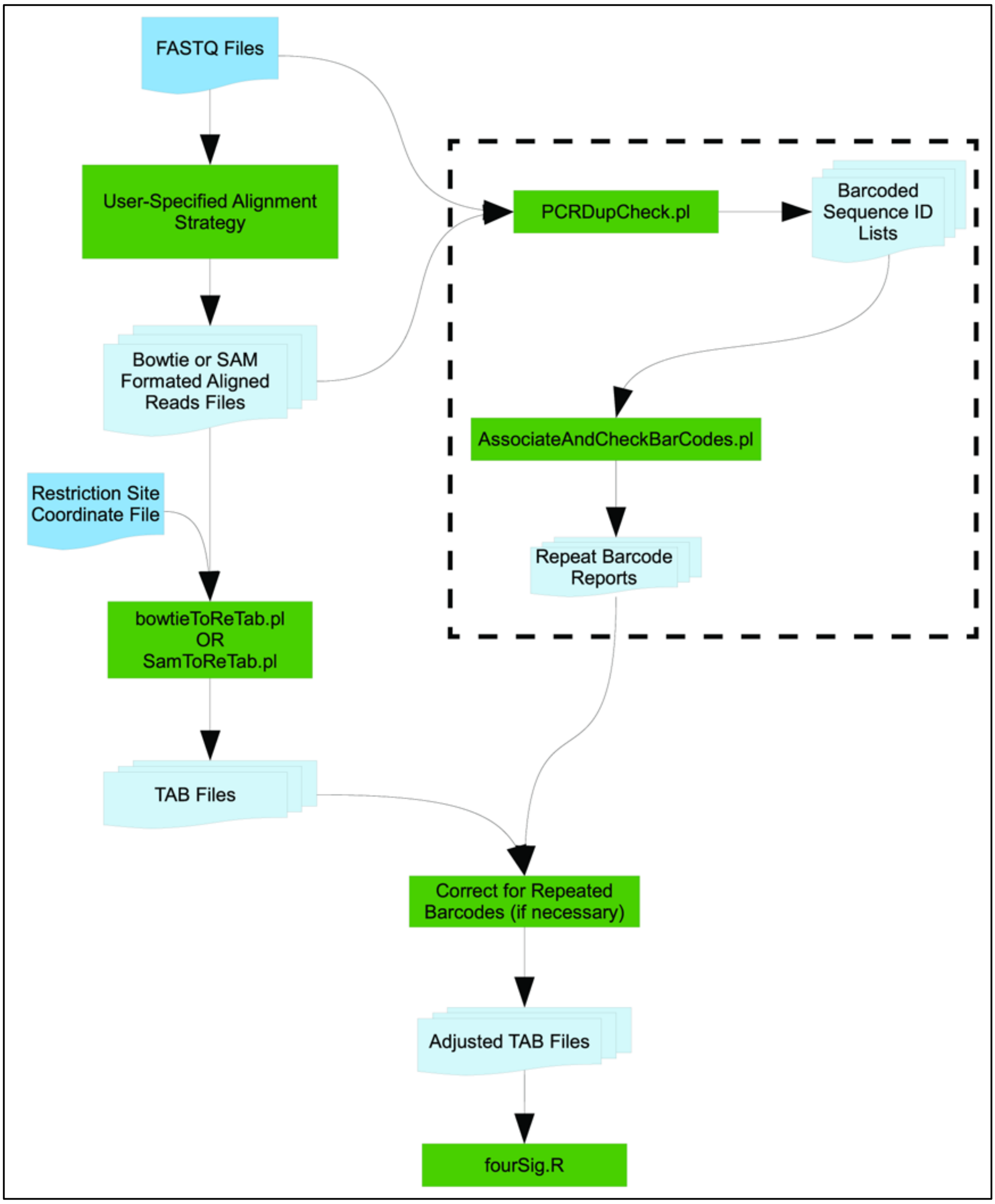

Figure S1. Preparation of Data for Analysis by *fourSig*. The schematic shows the process for taking raw sequence data (FASTQ) to the TAB file format that is used as an input for the *fourSig*. User supplied input files are represented by the dark blue, single page symbols. Specific programs or requirements for user action are represented by green rectangles. Program outputs are represented by the light blue multi-page symbols. For the *Ibtk* 4C data, amplification bias was assessed by analyzing read barcodes (dashed box). This step is not critical for formatting data to use the *fourSig* program.

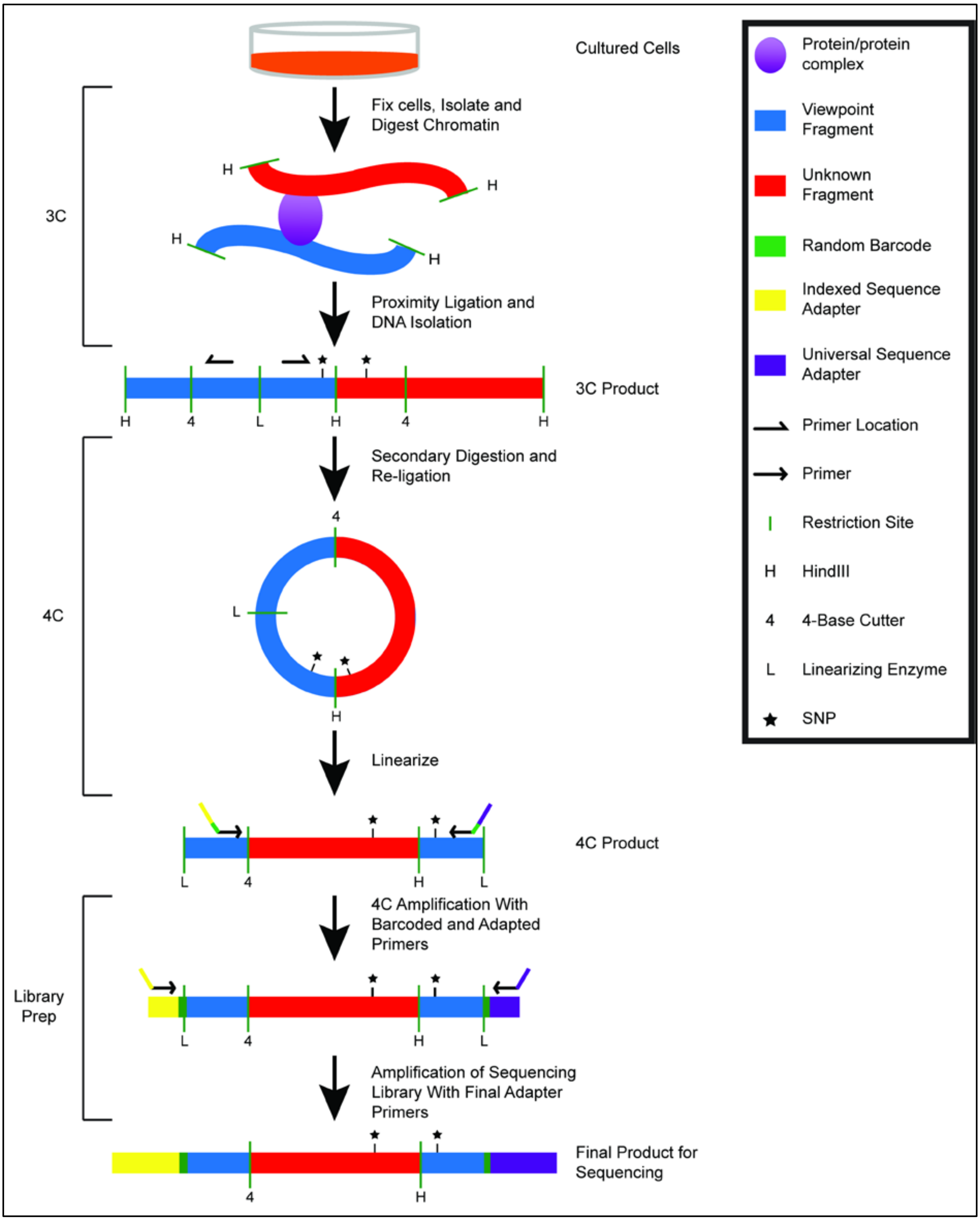

Figure S2. Process for Generating 4C-Seq Library. The schematic shows the process for generating allele-specific 4C-seq libraries. 3C libraries are first prepared from fixed cells using a 6-base cutter (H). Ligation products are shortened with by digestion with a 4-base cutter (4), re-ligated and purified to form circular products, and linearized with a 6 or 8-base cutter (L). Primers are designed against the desired viewpoint (blue), positioned to amplify the captured fragments (red). The 4-base cutter, linearizing enzyme, and positioning of the primers may be selected to capture a SNP (star) on the viewpoint fragment. Primers can be adapted to contain a randomized barcode (green) followed by the desired sequencing adapter (yellow, purple) to prepare the 4C library detection by high-throughput sequencing.

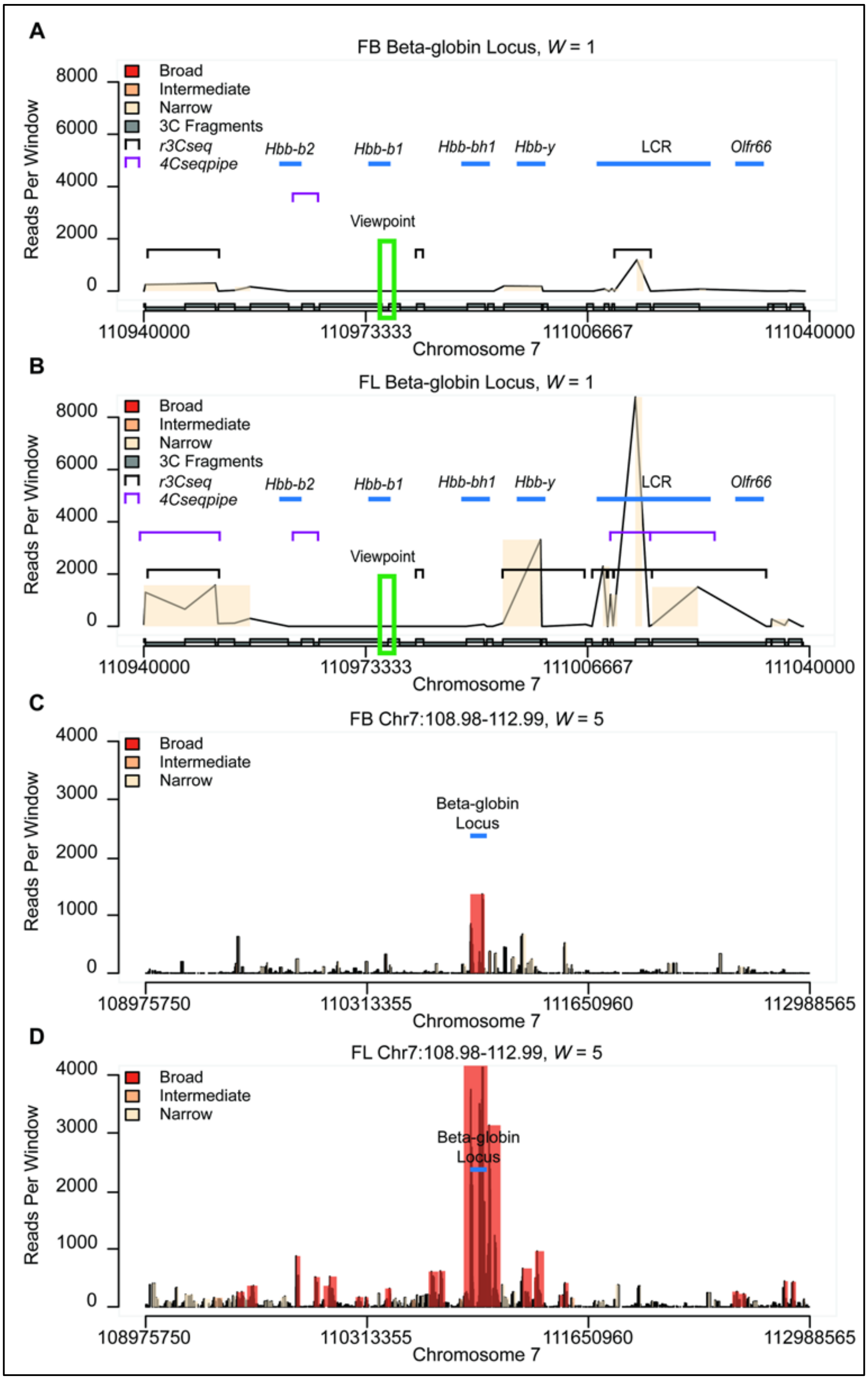

Figure S3. ***fourSig* analysis on 4C-seq data from the beta-globin locus.** Previously released 4C-seq data for the beta-globin locus from mouse fetal brain (A, C) and fetal liver (B, D) tissue were analyzed using *fourSig* as a benchmark for detecting enriched interactions. A-B. Interactions between the LCR and *Hbb-b1* are accurately detected in fetal liver tissue, but not the fetal brain, using a window size (*W*) of one 3C fragment. C-D. *fourSig* analysis of enriched *cis* interactions on Chromosome 7 within 2 Mb of *Hbb-b1* in both tissues is shown using a window size (*W*) of 5 fragments.

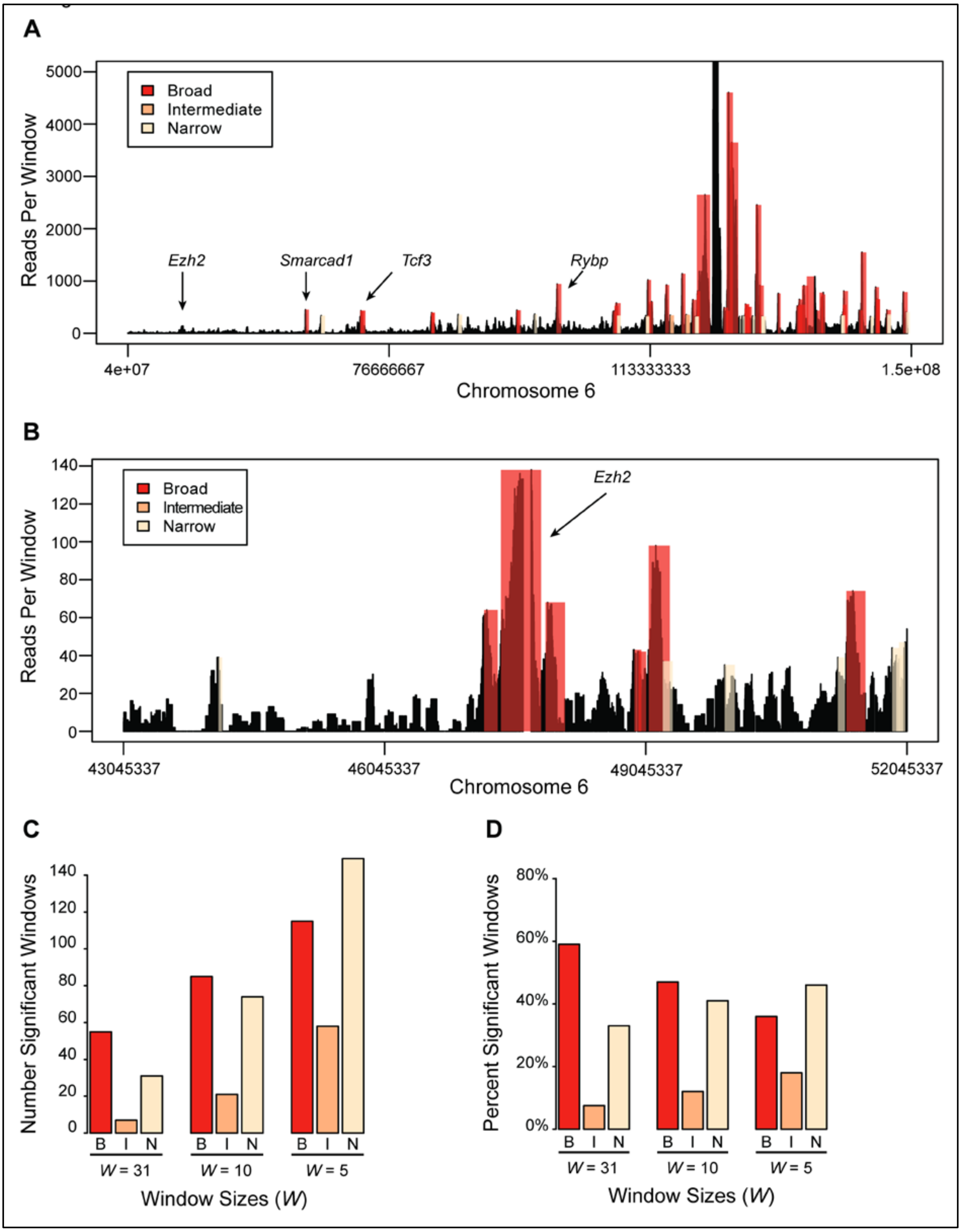

Figure S4. ***fourSig* Analysis of a *Nanog* 4C-Seq Dataset.** A. Previously described *cis*-interactions between *Nanog* and several genes on Chromosome 6 in mouse ES cells were detected with Broad classifications using the *fourSig* algorithm. B. Use of the masking feature to perform localized analysis near the *Ezh2* locus identifies a significant Broad interaction with the *Nanog* viewpoint. C. *fourSig* analysis calls more significant interactions as the window size is decreased. (B=Broad, I=Intermediate, N=Narrow). D. Decreasing the window size used for *fourSig* analysis results in a larger proportion of Narrow (N) interactions and a lower proportion of Broad (B) interactions.

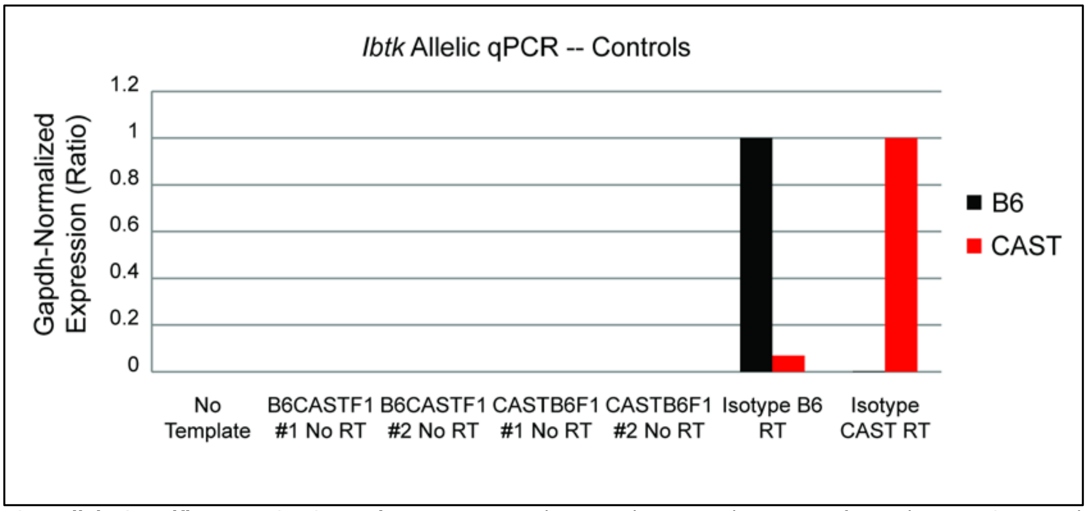

Figure S5. **Allele-Specific qRT-PCR Controls.** A. No RT and no template controls were run for each qRT-PCR experiment to verify that gDNA contamination was not detected. Additionally, cDNA prepared from allelic-isotype TS cells was tested to verify the allelic specificity of each amplicon. Results are relative to *Gapdh* expression and are not Log2 transformed.

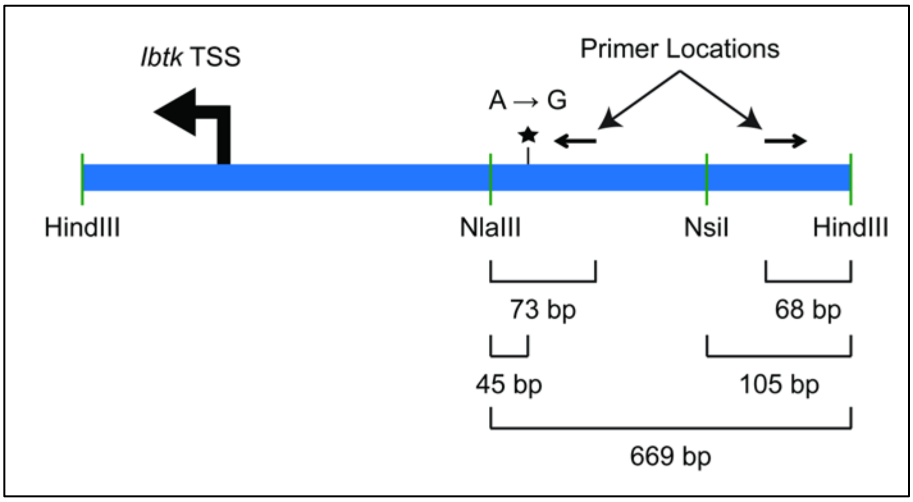

Figure S6. 4C Design at *Ibtk* Locus. The fragment containing the TSS for *Ibtk* (bent arrow) is depicted in the diagram. HindIII is used to create the initial 3C library. The 4C library is generated by secondary digestion with NlaIII and linearization with NsiI. Primers (straight arrows) were selected to capture the resulting unknown fragment (not shown). Allelic origin can be detected using the SNP (A in B6, G in CAST) that is captured on the NlaIII side by amplification. Genomic distances are shown below the diagram.

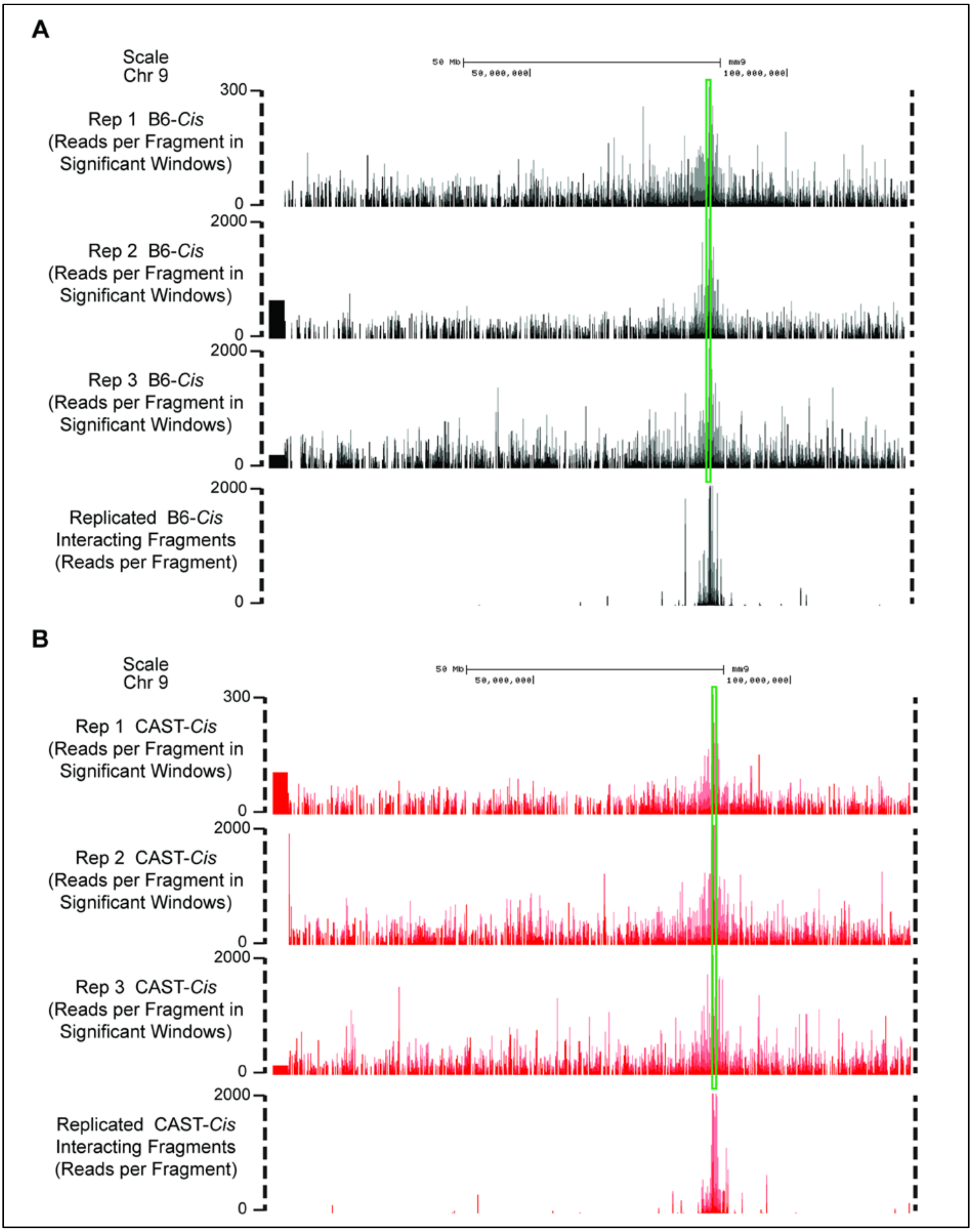

Figure S7. Replicated 4C Interactions and Intersecting Fragments. UCSC Genome Browser screenshots are shown for reads mapped to 3C fragments within *cis*-interactions (W = 5) for each replicate (first 3 tracks) and for the 3C fragments within these windows that were detected in all replicates (4th track). Interactions for the B6 allele (A) are shown in black and the CAST allele (B) are shown in red. A green rectangle is superimposed over the region containing the *Ibtk* gene. y-axis represents total reads detected.

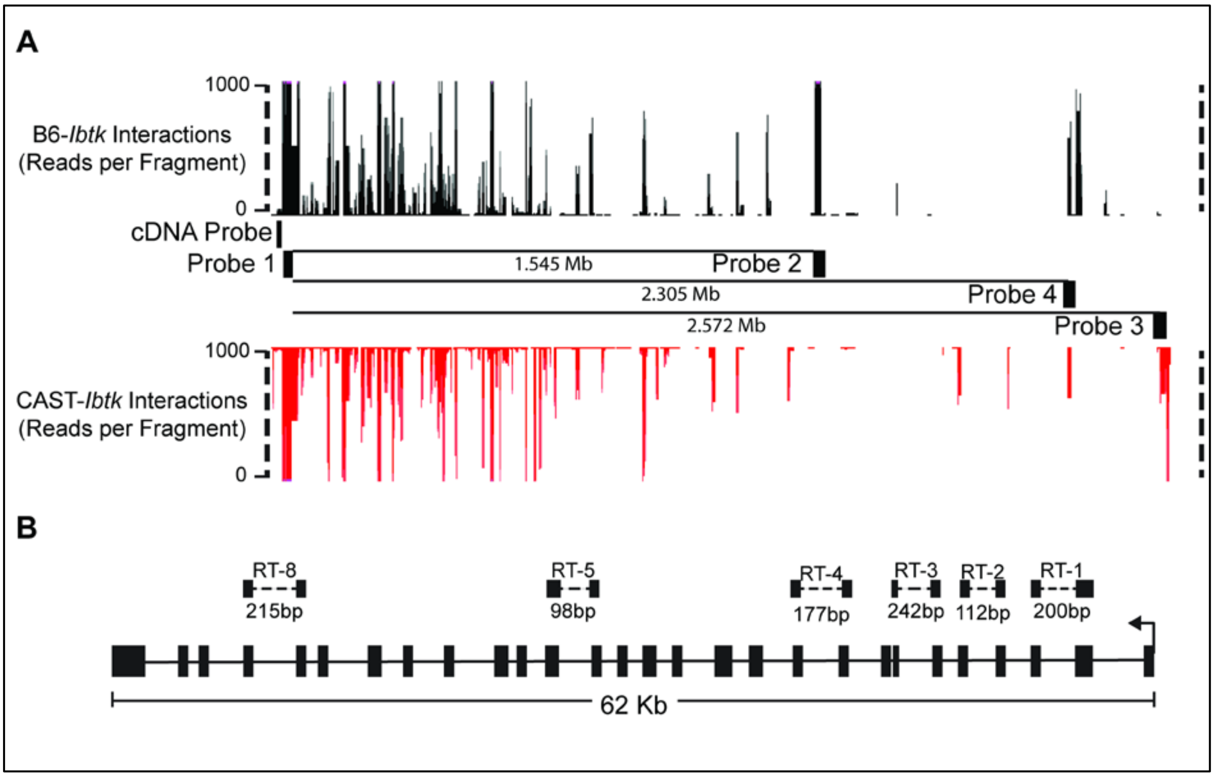

Figure S8. FISH Probe Locations. A. Fosmid probes were selected against a B6-specific interaction (2), a CAST-specific interaction (3), and a region with interactions common to both alleles (4). A fosmid probe covering the *Ibtk* TSS but upstream of the transcribed region was also selected to detect the gene locus (1). B. To detect *Ibtk* RNA, a mass-weighted cDNA probe was prepared by pooling purified RT-PCR product from six intron-spanning amplicons. The pooled probe contains just over 1 Kb of total DNA sequence.

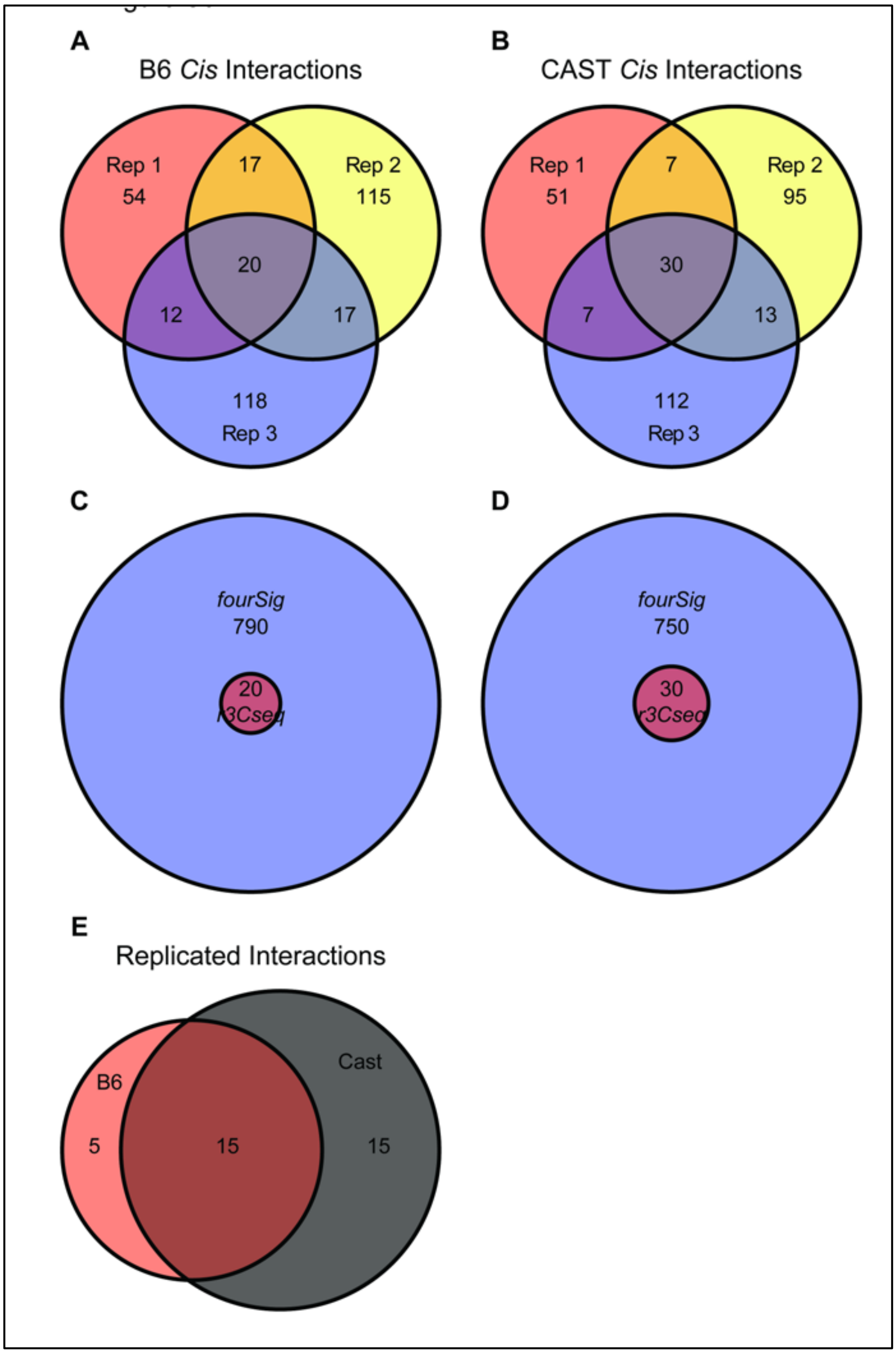

Figure S9. Allele-Specific Analysis of *Ibtk* 4C-Seq Data Using *r3Cseq*. A-B. Replicate intersections of fragments from interactions are displayed for (A) B6 and (B) CAST alleles. C-D. For both (C) B6 and (D) CAST alleles, all fragments consistently detected by *r3Cseq* were also detected by *fourSig.* E. As with the *fourSig* analysis, the majority of detected interactions were found in common between both alleles.

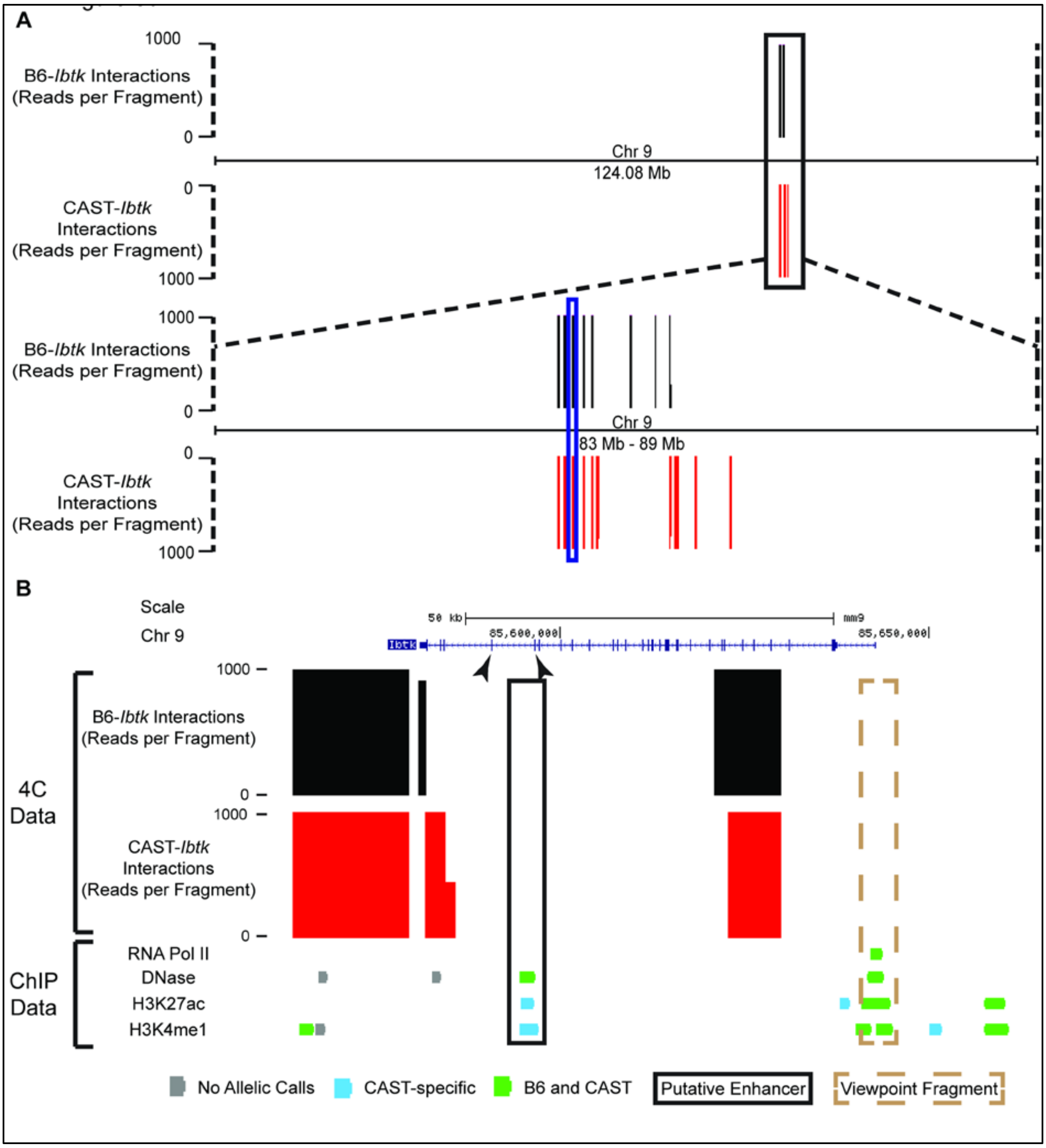

Figure S10. Distribution of *Ibtk* Interactions Detected by *r3Cseq*. A. Distributions of detected reads (black = B6 and red = Cast) for replicated, interacting fragments were plotted along Chromosome 9 (upper panel). The region surrounding the *Ibtk* locus (boxed) is expanded (lower panel). B. A UCSC Genome Browser screenshot of 4C interaction data and selected chromatin data at the *Ibtk* locus is shown. Exons 24 and 25 of *Ibtk* are highlighted with arrows. The 3C fragment containing the *Ibtk* TSS is indicated by a dashed brown rectangle. The location of the putative enhancer is enclosed by a solid black rectangle and its interaction is not detected by *r3Cseq*. For ChIP-Seq tracks, green boxes indicate biallelic enrichment, blue boxes indicate CAST-specific enrichment, and gray boxes indicate insufficient SNP detection to make an allele-specific call.

REFERENCES

1. Pfaffl,M.W. (2001) A new mathematical model for relative quantification in real-time RT-PCR. *Nucleic Acids Res.*, **29**, e45.

2. Meyer,L.R., Zweig,A.S., Hinrichs,A.S., Karolchik,D., Kuhn,R.M., Wong,M., Sloan,C.A., Rosenbloom,K.R., Roe,G., Rhead,B., et al. (2013) The UCSC Genome Browser database: extensions and updates 2013. *Nucleic Acids Res.*, **41**, D64–69.

3. Keane,T.M., Goodstadt,L., Danecek,P., White,M.A., Wong,K., Yalcin,B., Heger,A., Agam,A., Slater,G., Goodson,M., et al. (2011) Mouse genomic variation and its effect on phenotypes and gene regulation. *Nature*, **477**, 289–294.

4. Langmead,B., Trapnell,C., Pop,M. and Salzberg,S.L. (2009) Ultrafast and memory-efficient alignment of short DNA sequences to the human genome. *Genome Biol.*, **10**, R25.

5. Li,H., Handsaker,B., Wysoker,A., Fennell,T., Ruan,J., Homer,N., Marth,G., Abecasis,G., Durbin,R. and 1000 Genome Project Data Processing Subgroup (2009) The Sequence Alignment/Map format and SAMtools. *Bioinforma. Oxf. Engl.*, **25**, 2078–2079.
